# Therapeutic Potentials of Phytochemicals in Pancreatitis: Targeting Calcium Signaling, Ferroptosis, microRNAs, and Inflammation with Drug-Likeness Evaluation

**DOI:** 10.3390/nu17243841

**Published:** 2025-12-08

**Authors:** Fatma Farhat, Balaji Venkataraman, Bhoomendra A. Bhongade, Mauro Pessia, Shreesh Ojha, Sandeep B. Subramanya

**Affiliations:** 1 Department of Physiology, College of Medicine and Health Sciences, United Arab Emirates University, Al Ain P.O. Box 15551, United Arab Emirates or fatma_farhat@pharm.helwan.edu.eg (F.F.); mauro@uaeu.ac.ae (M.P.); 2 Department of Pharmacology and Toxicology, Faculty of Pharmacy, Helwan University, Ein Helwan 11795, Egypt; 3 Department of Pharmaceutical Chemistry, RAK College of Pharmaceutical Chemistry, RAK Medical & Health Sciences University, Ras Al Khaimah P.O. Box 15551, United Arab Emirates; bhoomendra@rakmhsu.ac.ae; 4 Department of Pharmacology and Therapeutics, College of Medicine and Health Sciences, United Arab Emirates University, Al Ain P.O. Box 15551, United Arab Emirates; shreeshojha@uaeu.ac.ae

**Keywords:** pancreatitis, acute pancreatitis, chronic pancreatitis, calcium signaling, ferroptosis, microRNAs, phytochemicals, Nrf2, NF-κB, drug-likeness, nanotechnology

## Abstract

Background: Pancreatitis, encompassing acute (AP), severe acute (SAP), and chronic (CP) forms, is a life-threatening inflammatory disorder with limited therapeutic options. Current management is largely supportive, highlighting the urgent need for novel interventions targeting underlying molecular pathways. Aim: This review summarizes recent advances in the pathogenesis of pancreatitis, focusing on calcium dysregulation, ferroptosis, and microRNA-mediated mechanisms while exploring the therapeutic potential of phytochemicals as disease-modifying agents. Summary: Aberrant calcium signaling, iron-dependent lipid peroxidation, and microRNA imbalance drive acinar cell injury, inflammatory cascades, and pancreatic fibrosis. Phytochemicals, including flavonoids, terpenoids, alkaloids, and phenolics, have shown protective effects in preclinical models through multi-targeted mechanisms. These include suppression of NF-κB-driven inflammation, activation of the Nrf2/HO-1 antioxidant pathway, modulation of ferroptosis via GPX4 and iron efflux, regulation of calcium signaling, and modulation of microRNA expression. Importantly, several phytochemicals attenuate acinar cell death, reduce cytokine release, and limit fibrosis, thereby improving outcomes in experimental pancreatitis. However, poor solubility, bioavailability, and pharmacokinetic limitations remain significant barriers. Emerging strategies such as nanotechnology-based formulations, prodrug design, and pharmacokinetic profiling, as well as bioavailability studies, may enhance their clinical applicability. Conclusions: Phytochemicals represent a promising reservoir of multitarget therapeutic agents for pancreatitis. Their ability to modulate oxidative stress, inflammatory and calcium signaling, ferroptosis, and microRNA networks highlights their translational potential. Future studies should focus on clinical validation, bioavailability optimization, and advanced delivery platforms to bridge the gap from bench to bedside.

## 1. Introduction

Pancreatitis is a serious medical condition characterized by inflammation of the pancreas. According to disease presentation and pathophysiology, it can be classified into acute, severe acute, and chronic pancreatitis. Acute pancreatitis (AP) is a sudden medical condition characterized by acute inflammation of the pancreas. It has different degrees of severity and can be associated with local or systemic complications. It is the leading cause of hospital admissions for gastrointestinal disorders in the USA and many other countries [1]. It is divided into two forms: necrotizing pancreatitis (NP) and interstitial edematous pancreatitis (IEP), according to the revised Atlanta classification. IEP is mild and self-limiting, resolves within 14 days, and can be effectively managed with supportive care. Nearly 5–10% of AP cases are NP, which is more severe and manifests as necrosis of the peripancreatic fat, pancreatic parenchyma, or both. The clinical episodes span from weeks to months, requiring extensive monitoring and intensive therapy because they can be associated with multiorgan failure [2]. Acute pancreatitis has an incidence of 110 to 140 per 100 people in the patient population, and around 80% of patients experience mild to moderate disease without organ failure. At the same time, 20% of patients exhibit severe acute pancreatitis (SAP), which involves multiorgan dysfunction and has a mortality rate of 20% [3]. Repeated acute episodes may progress to chronic pancreatitis, a progressive fibroinflammatory disease characterized by irreversible damage to pancreatic tissue, resulting in exocrine and endocrine dysfunction. The annual incidence of chronic pancreatitis is 10 cases per 100,000 in the general population [4].

Pancreatitis pathophysiology is complex, characterized by an interplay between molecular and cellular mechanisms. Starting with intracellular Ca^2+^ overload [5,6,7], which induces an inflammatory response and oxidative stress, leading to cell death [8,9]. Furthermore, excessive oxidative stress combined with iron overload triggers a chain of iron-dependent lipid peroxidation, inducing ferroptosis, a newly discovered form of cell death that has been recently shown to contribute to the pathogenesis of AP, SAP, and CP [10,11,12]. MicroRNAs are small, non-coding RNA molecules that regulate gene expression mainly through gene silencing [13,14] and, in some cases, induce gene transcription [15]. Increasing evidence suggests that microRNAs are involved in the development and progression of the three types of pancreatitis by modulating the expression of various harmful and cytoprotective proteins [16,17].

To date, the clinical intervention of AP, SAP, and CP depends on supportive care and symptomatic treatment, because no therapies have been formally approved. These interventions may be fluid resuscitation, nutritional support, analgesics, antibiotics, and enzyme replacement [18,19,20]. Thus, a deep understanding of the disease pathophysiology, pharmacotherapy development, and different therapeutic strategies is in demand.

Phytochemicals are naturally occurring bioactive compounds found in medicinal plants, herbs, and dietary sources that possess therapeutic potential across numerous diseases. These compounds, encompassing polyphenols, flavonoids, terpenoids, alkaloids, lignans, and saponins, have gained increasing attention as promising alternatives or adjuncts to conventional pharmaceuticals due to their multitarget mechanisms, minimal toxicity, and broad-spectrum biological activities [21,22]. More than half of all approved small-molecule drugs have originated directly or indirectly from natural products [23], demonstrating their enduring role in drug discovery and translational medicine.

In the context of pancreatitis, phytochemicals exhibit potent anti-inflammatory, antioxidant, anti-apoptotic, and antifibrotic effects through modulation of multiple signaling pathways implicated in pancreatic injury. Numerous preclinical investigations have revealed that compounds such as curcumin, resveratrol, quercetin, berberine, luteolin, and thymoquinone protect pancreatic acinar cells by attenuating oxidative stress, stabilizing intracellular calcium, and inhibiting key inflammatory mediators like NF-κB, MAPK, and COX-2 [24,25]. These agents also enhance endogenous antioxidant defense systems by activating NRF2/HO-1 signaling, thus restoring redox homeostasis and preventing lipid peroxidation and ferroptosis.

Furthermore, several phytochemicals regulate microRNA expression, thereby influencing post-transcriptional control of genes involved in inflammation, apoptosis, and fibrosis. For example, curcumin has been shown to upregulate miR-29a, suppressing TGF-β-mediated fibrogenic pathways, while berberine and resveratrol modulate miR-21 and miR-155, reducing NF-κB-driven cytokine release. Through such diverse mechanisms, phytochemicals exhibit pleiotropic effects that target multiple nodes within the pathological network of acute, severe acute, and chronic pancreatitis [26,27].

This review seeks to summarize the fundamental and recent molecular pathways in the pathogenesis of the three pancreatitis types, emphasizing the key role of calcium, ferroptosis, and microRNAs. Furthermore, we comprehensively summarize the proposed underlying mechanisms of phytochemicals and their protective effects against AP, SAP, and CP. Optimizing the therapeutic potential of phytochemicals and directing future drug development initiatives requires an understanding of the pharmacokinetic characteristics of the proposed compound. The drug-likeness and oral bioavailability of phytochemicals in our review were assessed by the combination of Lipinski’s Rule of Five (Ro5) and interactions with P-glycoprotein (Pgp), intestinal absorption, and Caco-2 permeability. Therefore, this review provides a reference for drug discovery and development in the treatment of pancreatitis.

## 2. Methodology

Electronic databases, including PubMed, Scopus, Google Scholar, and Google, were searched using keywords such as “pancreatitis”, “calcium”, “ferroptosis”, “microRNA”, “phytochemical”, “plant”, “natural product”, and “plant-based nutraceuticals”. This review focuses on studies conducted between 2005 and 2025 (spanning twenty years), utilizing both in vivo and in vitro experimental models of pancreatitis, as well as clinical studies. In addition to in vivo animal studies, several reports have employed in vitro models using isolated or immortalized pancreatic acinar cell lines to explore molecular mechanisms or the cytoprotective effects of phytochemicals. However, such in vitro systems do not replicate the complexity of pancreatitis, as they lack vascular, neural, and immune interactions and are not performed on the intact pancreas. Therefore, these experiments should be interpreted as assessing the direct damaging or protective effects of test compounds on neoplastic or immortalized pancreatic acinar cells rather than modeling the disease itself. It also highlights the potential benefits of phytochemicals and plant-based medicines for treating different types of pancreatitis. The sources of phytochemicals, target pathways, and reported effects are summarized in tables.

## 3. In Vivo Model of Pancreatitis

Various animal models of pancreatitis have been developed and utilized to better understand the disease’s pathogenesis and to identify new potential biomarkers that can predict disease onset and severity. Moreover, a solid understanding of the disease’s pathophysiology paves the way for discovering new therapeutic targets and treatments.

### 3.1. In Vivo Animal Models of Acute Pancreatitis

Although promising research has been conducted, no medication has been demonstrated to alter the course of acute pancreatitis. Consequently, the management of this condition is primarily supportive, and investigational therapies are not currently endorsed by existing guidelines. This is because our understanding of AP pathophysiology at the molecular level is limited, and the disease’s variable clinical progression, along with the challenge of studying the pancreas due to its location, makes research difficult. Therefore, several animal models have been developed to help clarify the underlying mechanisms and identify new biomarkers.

#### 3.1.1. Caerulein-Induced Acute Pancreatitis

This is a non-invasive method and is considered the most commonly used method for AP induction. Caerulein (CAE) is an analog of the cholecystokinin (CCK) hormone, which activates the CCK-A receptor in the acinar cells, inducing pancreatitis that resembles human edematous pancreatitis, exhibiting pathophysiological, morphological, and biochemical similarities to human pancreatitis [28]. Caerulein could be administered through intraperitoneal, subcutaneous, or intravenous injection at supraphysiological doses, 10–100 times above a physiological equivalent [29]. This method is reproducible, economical, and rapid. Additionally, CAE can be applied to in vitro models. At the same time, it has some drawbacks, such as the induction needing repeated injections and failing to replicate the severe features of human acute pancreatitis. Some studies combined CAE with lipopolysaccharide (LPS) or partial duct ligation to increase the severity [30,31].

#### 3.1.2. Alcohol-Induced AP (FAEE Model)

Fatty acid ethyl ester (FAEE) production is used to model alcohol-induced acute pancreatitis in rodents. Usually, ethanol is administered in combination with fatty acids like palmitoleic or oleic acid. By producing FAEEs that build up in the pancreas, this FAEE model causes acute pancreatitis by nonoxidative ethanol metabolism. These FAEEs lead to cytosolic Ca^2+^ overload, mitochondrial dysfunction, ATP depletion, and acinar cell necrosis, all of which are indicators of severe AP, while in vivo models involving ethanol plus POA reliably induce a severe, necrotizing pancreatitis phenotype [32]. In contrast to cerulein models, the FAEE model closely mimics the alcoholic form of AP in humans, especially in terms of systemic inflammation and mitochondrial damage. Mice and rats are commonly utilized to test protective medicines that target calcium signaling and oxidative stress pathways, as well as to examine mechanisms specific to ethanol [33].

#### 3.1.3. Basic Amino Acids

In rodents, necrotizing AP is caused by intraperitoneal administration of high concentrations of specific amino acids, such as L-arginine, L-ornithine, L-lysine, and L-histidine. To date, the most widely used amino acid is L-arginine-induced AP. Although its exact mechanism of action is yet unknown. It might be due to inhibiting protein synthesis, producing excessive nitric oxide, or causing lipid peroxidation [34]. Although several injections may occasionally be necessary, a single arginine injection is typically sufficient to cause acute necrotizing pancreatitis. The amount of arginine and the duration of exposure determine the severity of this animal model, and the main disadvantage is its lack of clinical relevance [35].

#### 3.1.4. Pancreatic Duct Ligation

Ligation of the common biliopancreatic duct results in backup of the pancreatic secretion and intraductal pressure with retention of the pancreatic enzymes within the PACs, leading to digestive enzymes, triggering inflammation and PACs injury. It can also be performed by obstruction of the pancreatic duct through insertion of a balloon-tipped catheter or vertical cannulation. The degree of damage depends on the duration of ligation. It initially presents with hyperamylasemia, inflammatory cell infiltration, and pancreatic edema, features consistent with acute pancreatitis. As time progresses, atrophy, loss of acinar cells, and fibrosis occur, which are compatible with chronic pancreatitis. The merits of this model are that it mimics the pathophysiology of gallstone-induced AP in humans, and it provides an invaluable way for studying the progression of the disease from acute to chronic. Importantly, in contrast to the chemical-induced model, the PDL method minimizes the off-target effects, allowing more accurate and specific investigations of disease pathogenesis. The disadvantage of this model is its technical difficulty, limited reproducibility, and rare use for AP due to its pathophysiological resemblance to chronic pancreatitis [35].

#### 3.1.5. Bile Acid Salts

The cannula is inserted through the biliopancreatic duct, and sodium taurocholate (NaT) or taurocholic acid (TCA) is infused in a retrograde manner, inducing severe hemorrhagic necrosis of the pancreas via direct damage to PACs. The features of the produced AP mimic biliary obstruction or gallstone-induced AP. Notably, the advantages of this model include a short induction period and the potential for pancreatic injury to be associated with multiorgan failure, which can be investigated, as well as a reproducible severity. The disadvantage is that laparotomy is required. The damage to the pancreas is uneven and is mainly limited to the main pancreatic duct surrounding area, leading to sampling error [35].

### 3.2. In Vivo Animal Models of Severe Acute Pancreatitis

#### 3.2.1. Two-Hit L-Arginine

Two intraperitoneal injections of L-arginine (4 g/kg) are administered to the mice with a one-hour gap between doses. Seventy-two (72) hours later, the identical operation is executed again. This leads to severe damage and extensive PAC necrosis, accompanied by significant production of proinflammatory cytokines such as TNF-α and IL-6, resulting in systemic inflammation and multiple organ dysfunction (MOD). This manifests as severe acute lung injury, renal impairment, and elevated intestinal permeability, facilitating a comprehensive comprehension of the commencement and therapeutic study of SAP [36].

#### 3.2.2. Sodium Taurocholate-Induced Severe Acute Pancreatitis

C57BL/6J mice are considered the gold standard for studying SAP pathophysiology, cytokine-driven inflammation, and therapeutic interventions because they exhibit severe disease features in a highly reproducible manner. The NaT model is an established method for SAP induction in C57BL/6J mice, which share significant clinical features of human biliary pancreatitis. Under general anesthesia, a 2.5% NaT solution is retrogradely infused into the common bile-pancreatic duct at a regular rate (10 µL/min for 3 min). Extensive PAC necrosis, pancreatic bleeding, and significant local and systemic inflammation appear within 12 to 24 h of infusion. In addition, the features of this model are MOD in the lung, kidney, and liver, evidenced by tissue histology and elevated levels of lipase, amylase, and IL-6 [37].

### 3.3. Animal Models of Chronic Pancreatitis

#### 3.3.1. Pancreatic Duct Ligation

The method of this model is described above. It is mainly used for inducing pancreatic fibrosis, which varies in its clinical and pathological features depending on the species. PDL in rats results in consistent PAC atrophy and fibrosis without significant inflammation. Unlike in rats, applying PDL in mice to produce uniform fibrosis is technically challenging because the mouse pancreas has three lobes: the splenic, duodenal, and gastric lobes that drain into separate ducts. However, this can be beneficial because it allows for internal controls, as it is easy to block only some of the pancreatic segments [38].

#### 3.3.2. Repeated Cerulein Injection

Human CP can result from repeated episodes of AP injury. Repeated doses of CAE-induced acute pancreatitis over several weeks cause chronic damage to the pancreas, leading to collagen deposits and fibrosis that mimic the pathogenesis of human CP. In brief, twice-weekly cerulein treatment at 50 μg/kg/h for 6 h over 10 weeks in mice induces acute reversible pancreatic injury, which eventually progresses to CP. This method is commonly used because of its ease and reproducibility [39].

#### 3.3.3. Alcohol-Induced Chronic Pancreatitis Model

Alcohol is one of the primary causes of CP. Several studies have reported that long-duration alcohol consumption alone does not induce an experimental model of CP. A combination of cerulein or lipopolysaccharide with alcohol resulted in fibrosis and a reduction of acinar cell mass [14]. In Sprague Dawley rats fed isocaloric Lieber-DeCarli liquid diets with alcohol for 10 weeks and challenged with a single dose or three repeated doses of the endotoxin lipopolysaccharide, stellate cell activation and fibrosis occurred [40].

#### 3.3.4. L-Arginine-Induced Chronic Pancreatitis

As described earlier, a single intraperitoneal injection of high-dose L-arginine (500 mg/100 mg body weight) induces severe necrotizing pancreatitis in rats. Interestingly, the long-term administration of L-arginine (350 mg/100 g body weight) for 4 weeks caused progressive degeneration of the pancreas. Only isolated single acinar cells could be seen within the fibrous connective tissue matrix contiguous with ducts, blood vessels, intrapancreatic nerves, and islets. This experimental model is simple to carry out and shows features similar to those of human chronic pancreatitis; however, the histological appearance is somewhat different [14].

## 4. Pancreatitis Pathophysiology

### 4.1. Calcium Signaling Dysregulation

#### 4.1.1. Physiological Calcium Signaling in Pancreatic Acinar Cells

**Figure 1:**
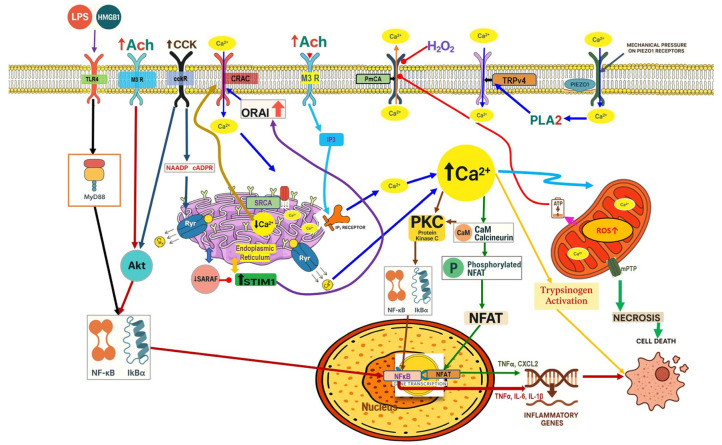
Calcium signaling in acute pancreatitis. The diagram summarizes key pathways contributing to pathological Ca^2+^ overload in pancreatic acinar cells. Activation of TLR4, CCK, and cholinergic receptors triggers IP_3_- and NAADP-dependent Ca^2+^ release from the endoplasmic reticulum, followed by STIM1–Orai1-mediated store-operated Ca^2+^ entry. Mechanical and oxidative stimuli activate PIEZO1 and TRPV4 channels, further increasing cytosolic Ca^2+^ levels. Persistent Ca^2+^ elevation activates PKC and calcineurin–NFAT signaling, enhances NF-κB nuclear translocation, and induces transcription of inflammatory cytokines. Mitochondrial Ca^2+^ overload promotes ROS generation, mPTP opening, ATP loss, trypsinogen activation, and necrotic cell death. These coordinated events drive inflammation and tissue injury in acute pancreatitis.

PACs comprise approximately 80% of the exocrine pancreas mass, where the digestive proenzymes are synthesized and stored in zymogen granules. These cells exhibit distinct basal and apical regions, with the latter containing the zymogen granules. Calcium signaling plays a crucial role in regulating digestive enzyme secretions from PACS, which occur via the stimulation of two receptors, the muscarinic M3 receptor (M3) and the CCK1 receptor. Acetylcholine activates M3 receptors, inducing a downstream signal pathway that leads to the production of inositol trisphosphate (IP3), which stimulates the IP3 receptor (IP3R), leading to the release of Ca^2+^ from the ER, triggering transient Ca^2+^ elevation localized in the apical region, followed by zymogen exocytosis from the apical area. On the other hand, the activated CCK1 receptor stimulates the production of cyclic ADP-ribose (cADPR) and nicotinic acid adenine dinucleotide phosphate (NAADP), which mediate calcium release from the ER through the ryanodine receptor (RyR). ER-released Ca^2+^ enters the mitochondrial matrix via the mitochondrial Ca^2+^ uniporter (MCU), where it is used for ATP production, and excess Ca^2+^ returns to the cytoplasm via the Na^+^/Ca^2+^ exchanger (MNCX) [41]. The depletion of ER Ca^2+^ caused by excessive release through ryanodine receptors (RyR) and inositol-1,4,5-trisphosphate receptors (IP_3_R) activates store-operated Ca^2+^ entry (SOCE). The loss of luminal Ca^2+^ is sensed by STIM1, which primarily activates ORAI1 channels on the plasma membrane, allowing Ca^2+^ influx for ER refilling; TRPC channels may contribute to a lesser extent. The newly entered Ca^2+^ is then pumped back into the ER by Sarco/Endoplasmic Reticulum Ca^2+^-ATPase (SERCA) to restore luminal Ca^2+^ levels. When the endoplasmic reticulum’s Ca^2+^ levels drop, Stromal Interaction Molecule 1 (STIM1), which is an ER membrane Ca^2+^ sensor, polymerizes and attaches to Orai1 to create SOCE channels that transport Ca^2+^ into the cell, such as the Orai1/Ca^2+^ release-activated Ca^2+^ channel (CRAC) [42,43]. Following ER Ca^2+^ refilling, the cytosolic Ca^2+^ is elevated. Then, the ER membrane protein called the SOCE-associated regulatory factor (SARAF) inactivates Orai1 to avoid uncontrolled Ca^2+^ influx [44,45]. Excessive cytosolic Ca^2+^ is pumped out of the cell by the plasma membrane Ca^2+^ ATPase (PMCA) [46] (Figure 1).

#### 4.1.2. Calcium Signaling Dysregulation in Pancreatitis

Aberrant intracellular Ca^2+^ signaling represents a pivotal event in pancreatitis, initiating premature trypsinogen activation, mitochondrial dysfunction, and necrotic cell death. In acinar cells, excessive Ca^2+^ release via inositol 1,4,5-trisphosphate receptors (IP_3_R) and ryanodine receptors (RyR) depletes ER stores, thereby activating store-operated Ca^2+^ entry (SOCE) primarily via Orai1 channels, with TRPC channels contributing to a lesser extent. The refilling of ER Ca^2+^ via coupling of Orai1 with STIM1 and SERCA occurs in the absence of a measurable cytosolic Ca^2+^ rise [47,48,49]. While under physiological conditions, SOCE-associated regulatory factor (SARAF) terminates Orai1 activity once refilling is complete, and its downregulation in acute pancreatitis amplifies Ca^2+^ influx and tissue injury [44,45,50]. Sustained cytosolic Ca^2+^ overload triggers NF-κB and NFATs activation [51,52,53], mitochondrial permeability transition, ATP depletion, and necrosis [54] (Figure 1).

SOCE through ORAI1 is the central Ca^2+^ signaling mechanism integral to many neutrophil functions, such as degranulation, leukotriene synthesis, and ROS production [55,56]. Neutrophil-specific Orai1-deficient mice were protected against pancreatitis-associated acute lung injury [57]. Under normal physiological conditions, ER is physically connected to specific mitochondrial membrane parts through a macromolecular complex, IP3R-Grp75-VDAC1, which facilitates the transfer of Ca^2+^ from ER to mitochondria, where Ca^2+^ is used for ATP production. Voltage-dependent anion channel 1 (VDAC1), localized in the outer mitochondrial membrane, facilitates Ca^2+^ entry to mitochondria. IP3R is found in the ER membrane through which Ca^2+^ is released. Grp75 tethers the *N*-terminal domain of the IP3R to VDAC1, stabilizing the coupling of IP3R and VDAC1 and improving Ca^2+^ transfer from the ER to mitochondria [58]. Recently, a study of SAP in mice demonstrated that the ER–mitochondria Ca^2+^ transport increased in the pancreatic tissue and was observed by elevating levels of the protein of calcium channel IP3R-VDAC1, which leads to mitochondrial Ca overload [59], followed by mitochondrial membrane potential loss, ATP depletion, and cell necrosis [60] (the figure in Section 4.4).

### 4.2. Ferroptosis

Ferroptosis is a new type of regulated cell death. It was first identified in 2012 [61]. Increasing evidence has discovered its contribution to the pathogenesis of a broad spectrum of diseases. Contrasting with apoptosis, autophagy, and necrosis, ferroptosis is an iron-dependent lipid peroxidation of membrane phospholipids [62]. Ferroptosis is considered an interconnected process, starting with iron overload concurrently with compromised antioxidant defenses, resulting in uncontrolled lipid peroxidation of polyunsaturated fatty acids (PUFAs) in membrane phospholipids, which leads to plasma membrane permeabilization, rupture, and cell death [63]. It has three phases: initiation, propagation, and termination. The initiation of PL peroxidation includes both enzymatic and nonenzymatic pathways, followed by sequential lipid autoxidation, which propagates the ferroptotic signal. Antioxidant systems can terminate this autooxidation process [64].

Acyl-CoA synthetase long-chain family member 4 (ACSL4) converts long-chain PUFAs such as arachidonic acid and adrenic acid into acyl-CoA forms, and then LPCAT3 helps turn these into PUFA-PE substrates, which are then integrated into the membrane phospholipid [65]. These PUFA-PE molecules contain more than one double bond and bis-allylic moieties, which make them liable to oxidation by enzymes such as ALOX and POR and nonenzymatic reactions (e.g., using an iron-dependent Fenton reaction), forming PL hydroperoxides (PL-PUFA-OOHs), a central pro-ferroptotic signal [66,67]. Iron plays a pivotal role in the ferroptosis process through enhancing the enzymatic and nonenzymatic lipid peroxidation. Regarding the enzymatic, iron acts as a catalyst for ALOX and POR enzymes. In addition, ferrous iron initiates the Fenton reaction, in which it interacts with hydrogen peroxide, forming a highly mobile and toxic form of ROS, hydroxyl radicals (OH•), which interact with PUFA-PLs to initiate and propagate ferroptosis [68].

On the other hand, impairment of antioxidant systems makes cells vulnerable to ferroptosis. Two antioxidant systems can counteract the ferroptosis mechanism; the primary one is the XC-GSH-GPX4 system, along with GPX4-independent mechanisms [69].

#### Ferroptosis Role in Pancreatitis

**Figure 2:**
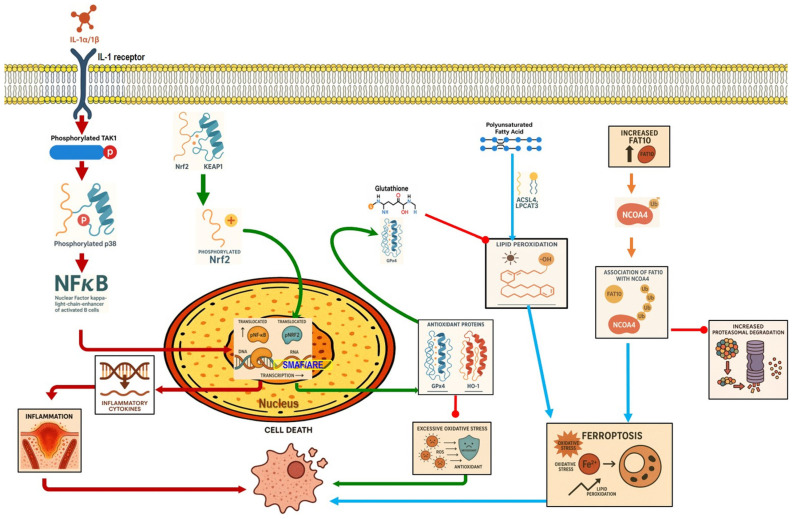
p38 MAPK/Nrf2/ferroptosis signaling in acute pancreatitis. This figure illustrates the interaction between IL-1α/β-induced p38 MAPK/NF-κB activation and the Nrf2 antioxidant pathway in regulating oxidative stress, lipid peroxidation, and ferroptosis. The binding of IL-1α/IL-1β to its receptor activates TAK1 and p38, promoting NF-κB nuclear translocation and transcription of proinflammatory cytokines. Under oxidative conditions, KEAP1 dissociation enables Nrf2 phosphorylation and nuclear translocation, driving expression of antioxidant genes, including GPX4 and HO-1. Polyunsaturated fatty acids, activated by ACSL4 and LPCAT3, undergo lipid peroxidation when GPX4 activity or glutathione availability is reduced. FAT10 accumulation enhances NCOA4 ubiquitination, promoting proteasomal degradation and dysregulation of ferritin turnover. These events increase free Fe^2+^, amplify reactive oxygen species, and drive ferroptotic cell death. The combined inflammatory signaling, impaired antioxidant defense, and lipid peroxidation contribute to cellular injury and inflammation.

Increasing evidence has reported that ferroptosis contributes to the AP pathophysiology [10,11]. Expression of human leukocyte antigen (HLA)-F adjacent transcript 10 (FAT10) is increased as AP progresses. As a result of its competition with ubiquitin for binding to nuclear receptor coactivator 4 (NCOA4), it inhibits ubiquitination and forms a stable FAT10-NCOA4 complex that is resistant to proteasomal degradation. Consequently, pancreatic acinar cells undergo ferroptosis due to elevated NCOA4 expression [70]. In addition, an acute HTGP study demonstrated that the pancreas exhibited upregulation in ACSL4 and LPCAT3, accompanied by a significant decrease in GPX4 and xCT [71] (Figure 2).

**Figure 3:**
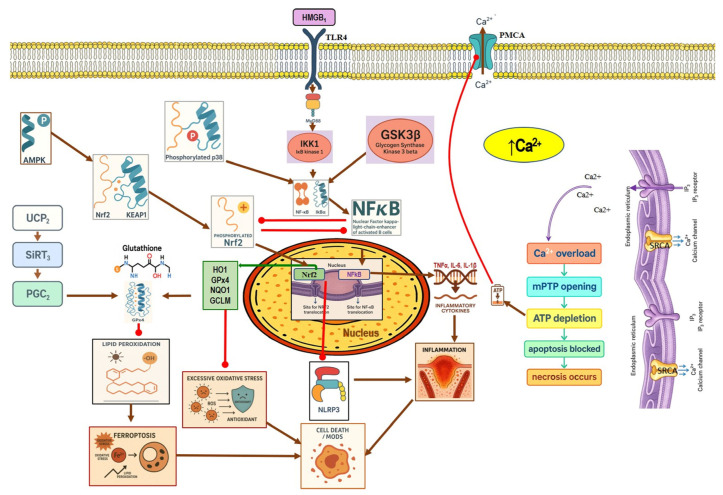
Severe acute pancreatitis pathophysiology. This figure depicts key molecular events linking metabolic stress, inflammatory signaling, and Ca^2+^ dysregulation in severe acute pancreatitis. HMGB1–TLR4 activation triggers MyD88-dependent signaling through IKK1, GSK3β, and p38 MAPK, promoting NF-κB nuclear translocation and the transcription of proinflammatory cytokines (TNF-α, IL-1β, IL-6). AMPK activation facilitates Nrf2 phosphorylation and KEAP1 dissociation, inducing HO-1, GPX4, NQO1, and GCLM expression and enhancing antioxidant defense. Impaired glutathione–GPX4 activity and increased lipid peroxidation drive ferroptosis, while excessive oxidative stress activates the NLRP3 inflammasome, contributing to systemic inflammation and cell death. Elevated cytosolic Ca^2+^ promotes ER Ca^2+^ release via IP_3_ receptors, mitochondrial Ca^2+^ overload, mPTP opening, ATP depletion, and failure of apoptotic clearance, culminating in necrosis. The combined effects of inflammatory cytokines, redox imbalance, ferroptosis, and Ca^2^-dependent mitochondrial injury exacerbate tissue destruction and multiorgan dysfunction.

Recent studies have implicated that ferroptosis plays a key role in SAP pathogenesis. A study of SAP-ALI has proven that knocking down uncoupling protein-2 (UCP2) uncouples oxidative phosphorylation from ATP production and plays a crucial role in ROS handling, enhances ferroptosis, and ROS production. The activation of the UCP2-SIRT3-PGC1α signaling pathway suppresses ferroptosis by maintaining the expression of GPX4, a key protein that prevents lethal lipid peroxidation [72] (Figure 3). Similarly, in another SAP study, ferroptosis-related proteins were upregulated, showing autophagy can enhance the ferroptosis process [73]. The SAP pancreatic tissue of mice exhibits significant glycolytic abnormalities, and the elevated lactate level resulting from glycolytic abnormalities further enhances the histone acetylation in the HIF1A promoter region, thereby aggravating the expression of HIF1A. Lactate-dependent HIF1A transcriptional activation exacerbates severe acute pancreatitis through the ACSL4/LPCAT3/ALOX15 pathway, inducing ferroptosis [74].

Recent studies reveal that ferroptosis contributes to CP progression. GPX4 deletion in acinar cells under chronic alcohol exposure intensified pancreatic fibrosis, inflammation, and lipid peroxidation, while treatment with the ferroptosis inhibitor olanzapine alleviated these effects [75]. Chronic pancreatitis features were shown in iron-overloaded mice, which exhibited a downregulation of anti-ferroptosis proteins, such as SLC7A11 and GPX4, and an elevation in the pro-ferroptosis proteins, such as cytochrome c oxidase subunit II [76].

### 4.3. Nrf2 Role in Pancreatitis

Nuclear factor erythroid 2-related factor 2 (Nrf2) is a transcriptional factor that has a crucial role in maintaining the cell redox homeostasis through regulating the expression of several antioxidant and phase 2 enzymes. In the cytosol, Nrf2 binds to Kelch-like ECH-associated protein (Keap1), which negatively regulates Nrf2 via enhancing its proteasomal degradation [77,78].

In various AP models, the activation of Nrf2 and heme oxygenase-1 (HO-1) signaling pathways has been shown to mitigate oxidative stress and inflammation [79]. There is a known interaction between oxidative stress and inflammation, where the inflammatory response triggered by excessive reactive ROS generation is mediated by the transcription factor NF-Κb. Nrf2 inhibits NF-Κb signaling by promoting the degradation of the IκB proteins [79,80]. Mouse embryonic fibroblasts deficient in Nrf2 exposed to LPS showed an increase in IKKβ activity, leading to phosphorylation and degradation of IκBα. Resulting in enhanced NF-Κb activation [81]. Nrf2 expression was decreased in PAC treated with CAE, while markers of inflammation, such as NF-κb, IL-6, TNF-α, and nitrotyrosine, were elevated [27] (Figure 2).

Several studies have reported that the SAP group’s pancreatic and lung tissues showed a decrease in Nrf2 and HO-1 expression. Gao et al. conducted a study of AP-associated lung injury. They proved that activation of Nrf2/HO-1 in the lung tissue suppressed the ROS-induced NLRP3 inflammasome, which enhanced the production of the proinflammatory cytokines and caspase-1 activation [82]. Nrf2-knockout mice were more vulnerable to cellular death than wild-type mice after caerulein administration plus LPS [83]. Furthermore, according to Matzinger et al., AMPK phosphorylates Nrf2 at serine 374, 408, and 433, then Nrf2 translocates from the cytoplasm to the nucleus and binds to the ARE gene to produce its antioxidant effect [84]. Rats treated with AMPK agonists demonstrated that AMPK plays a crucial role in triggering antioxidant effects mediated by hepatic Nrf2 signaling, which protects against pancreatitis-related liver damage induced by sodium taurocholate and L-arginine [58] (Figure 3).

In addition, several research outcomes have demonstrated that increased Nrf2 and HO-1 signaling activation in SAP models can reduce oxidative stress and inflammation [85,86,87]. Several models of SAP mice showed the crosstalk between Nrf2/NF-κb p65 signaling pathways, and this appeared as Nrf2 downregulation and NF-κb p65 activation in the pancreas of SAP mice, which increased cytokine production, inducing inflammation [88,89,90] (Figure 3).

**Figure 4:**
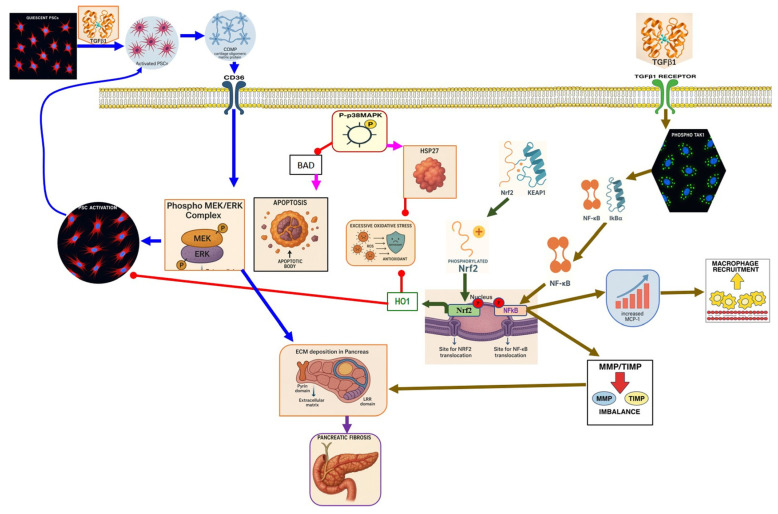
Chronic pancreatitis pathophysiology. This figure illustrates key mechanisms linking CD36 signaling, TGFβ1 activation, oxidative stress responses, and MAPK pathways to pancreatic stellate cell (PSC) activation and fibrogenesis. Quiescent PSCs are activated by TGFβ1 and COMP, leading to CD36 engagement and downstream MEK/ERK phosphorylation, PSC proliferation, and apoptosis. CD36 signaling enhances extracellular matrix (ECM) deposition, contributing to pancreatic fibrosis. Concurrently, TGFβ1 receptor signaling activates TAK1 and promotes NF-κB translocation, increasing MCP-1 expression and macrophage recruitment. Elevated oxidative stress triggers p38 MAPK phosphorylation and HSP27 activation, while Nrf2–KEAP1 dissociation allows Nrf2 nuclear translocation and HO-1 induction. Imbalance between Nrf2-mediated antioxidant defenses and NF-κB-driven inflammation amplifies ECM accumulation, MMP/TIMP dysregulation, and progressive fibrotic remodeling in chronic pancreatitis.

Recently, a study demonstrated that activation of the Nrf2/HO-1 signaling pathway reduces oxidative stress and downregulates fibrotic markers such as Col1a1, Col4a1, and α-SMA, thereby inhibiting PSC activation and extracellular matrix deposition [26] (Figure 4).

### 4.4. NF-κB in Pancreatitis

Nuclear factor Kappa B (NF-κB) is a transcriptional factor that regulates the expression of different genes, such as inflammatory genes and genes responsible for tissue injury and repair. During unstressed circumstances, NF-κB binds to its inhibitory regulator, IκB. However, exposure to stimulating factors, such as ROS and cytokines, phosphorylates the IκBs, triggering their proteasomal degradation. After that, NF-κB is activated and translocates to the nucleus, inducing transcription of specific genes [91].

In the context of AP, NF-κB is activated by ROS, non-physiological calcium overload, and activated protein C isoforms [92]. A study showed that activation of calmodulin/calcineurin signaling by calcium results in enhanced NF-κB nuclear translocation, as evidenced by a reduction in the NF-κB activity after inhibition of calcineurin [93,94]. Furthermore, PKC constitutes a family of serine/threonine kinases, several of which are critical upstream mediators of NF-κB. A study proved that PKC activation by Ca^2+^ is essential for NF-κB translocation. This was shown through using Ca^2+^ chelators such as BAPTA, which resulted in a reduction in PKC phosphorylation, IkB-α phosphorylation, and degradation [8,9]. Additionally, there is a crosstalk between the calcineurin pathway and PKC, where calcineurin activates NF-κB through PKC-δ activation, resulting in cytokine production and acinar cell death [93]. CCK-A receptors and cholinergic receptors are G-protein-coupled receptors found on the cell surface in the PACs. Samuel et al. reported that ligation-induced AP in mice hyperstimulates these receptors, which activate the Akt protein. Activated Akt enhances transcriptional activity of NF-κB, elevating the production of inflammatory mediators [95] (Figure 1). In addition, one of the pivotal inflammatory signaling pathways is the TLR4/NF-κB pathway, which is implicated in the AP-related inflammatory process. TLR4 activation induces MyD88, which recruits TRAF6; subsequently, numerous downstream proteins are activated, ultimately leading to the induction of the NF-κB signaling, contributing to the AP initiation and progression [96]. Several research findings have revealed that the TLR4/NF-κB signaling pathway is involved in the experimental AP pathogenesis [97,98] (Figure 1).

In the context of SAP, harmful stimuli activate TLR4, which triggers the NF-κB signaling pathway [99]. Li et al. demonstrated that TLR4 was upregulated by the rhHMGB1, resulting in NF-κB signaling induction, aggravating the pancreatic tissue injury in SAP mice [100]. Furthermore, GSK-3β phosphorylates IKK-β, which leads to the translocation and activation of NF-κB [101]. Thus, the pharmacological inhibition of GSK-3β reduced the NF-κB activity and showed a protective effect in renal inflammatory changes associated with the SAP [102] (Figure 3). Numerous clinical and preclinical studies revealed that NF-κB plays a crucial role in CP development and progression. Progressive fibrosis is considered the final pathological change in CP, and PSC activation is an early cellular event in initiating pancreatic fibrosis. Previous research has shown that TGF-β1 regulates PSC activation [103]. In the context of pancreatic inflammation, overexpressed TGF-β1 stimulates the TGF-β receptor-1 on PSCs, activating the NF-κB signaling pathway. NF-κB activation further promotes fibrosis and accelerates pancreatic inflammation [104]. Additionally, pancreatic tissue samples from patients with CP show increased activation of NF-κB compared with controls [105] (Figure 4).

### 4.5. p38 MAPK in Pancreatitis

The MAPK signaling pathway is a serine/threonine protein kinase family that contains three main proteins: p38 MAPK, extracellular regulated protein kinases (ERKs), and c-Jun *N*-terminal kinase (JNK) [106]. The stress-responsive MAPK is p38 MAPK, which plays a fundamental role in intracellular signal transduction, and oxidative stress and inflammatory factors can activate it [107]. Activated p38 modulates several downstream targets such as transcription factors, cytoskeletal and scaffold proteins, metabolic enzymes, and molecular chaperones [108]

The p38 MAPK pathway has a key role in the pathogenesis and progression of AP. Once it is activated, it exacerbates the inflammation process via the production of inflammatory cytokines. Administration of CAE and LPS to induce AP in mice increases serum IL-1β and IL-1 that activate IL-1R, increasing TAK protein phosphorylation, activating p38MAPK, and causing inflammatory effects and cell apoptosis, evidenced by the elevation of cleaved Caspase-3 level [109]. Moreover, treatment of the PAC with TNF-α and CCK leads to p38 MAPK activation, resulting in phosphorylation of downstream protein kinase MK2, activating the NF-κB inflammatory pathway [110] (Figure 2).

Growing evidence proves that the p38MAPK signaling pathway is involved in developing SAP. Inhibition of p38MAPK increases HO-1, which possesses cytoprotective properties, including anti-inflammatory, antioxidant, and anti-apoptotic activities, and contributes to the inhibition of pulmonary proinflammatory and pro-apoptotic signaling pathways in SAP with ALI [111]. Zhang et al. proved that inhibition of the P38 MAPK signaling pathway showed an anti-inflammatory effect, which protects against renal injury associated with ANP [112] (Figure 3).

Several studies have demonstrated that p38MAPK plays a significant role in the pathogenesis of CP. p38MAPK operates in a cell-type-specific and cell-context-specific manner to integrate signals that impact cell survival, differentiation, and proliferation [113]. The MAPK pathway has shown a controversial role in CP, as some studies have found that it can act as a protective mechanism or contribute to pathogenesis. It has been reported that p38MAPK alleviates apoptosis and oxidative stress during CP by downregulating BAD expression and upregulating HSP27 expression. BAD is a pro-apoptotic protein belonging to the Bcl-2 family; it binds to and ultimately inactivates anti-apoptotic family members such as Bcl-2 and Bcl-xL. p38 MAPK inhibition promotes BAD expression in the pancreas, reduces the levels of the downstream target antioxidant protein HSP27 in the pancreas, and leads to cell death [114]. In contrast, another study showed that p38MAPK is expressed in activated PSCs, reflecting its role in the pancreatic fibrosis of CP mice, and that ERK and JNK were directly involved in the activation of PSCs induced by TGF-β1 and the development of pancreatic fibrosis [103]. Activated PSCs play an important role in the progression of pancreatic fibrosis, and inhibiting the phosphorylation of p38 MAPK, ERK1/2, and JNK1/2 of PSCs showed an anti-fibrotic effect [115]. In addition, activated PSCs by TGF-β1 produce cartilage oligomeric matrix protein (COMP) into the extracellular environment, which stimulates the PSCs’ surface receptor CD36, followed by activation of the MEK/ERK pathway, triggering the activation and proliferation of PSCs and the production of ECM collagen-I. These activated PSCs also activate paracrine or autocrine COMP, increasing the activation of nearby PSCs or themselves, creating a vicious cycle that eventually results in sustainable and progressive pancreatic fibrosis [116] (Figure 4).

### 4.6. MiRNA Role in Pancreatitis

MicroRNAs are small, non-coding RNA molecules involved in various physiological and pathological processes, including cell growth, development, and death [117]. Mainly, they regulate the post-transcriptional gene expression. Most studies to date have shown that miRNAs bind to a specific sequence at the 3′ UTR of their target mRNAs to induce translational repression and mRNA deadenylation and decapping [13,14]. Furthermore, miRNA interaction with the promoter region has been reported to induce transcription [15].

Several preclinical and clinical studies have shown that miRNAs are implicated in the AP pathogenesis. L-arginine-induced acute edematous pancreatitis in rats exhibited a significant upregulation of miR-135a and miR-22 in the pancreatic tissues. The elevated miR-135a and miR-22 target and block the expression of erb-B2 receptor tyrosine kinase 3 (ErbB3) and protein tyrosine kinase 2 (Ptk2), respectively, leading to PACs apoptosis [118]. In addition, miRNAs exert a regulatory role in both autophagy and necrosis in different experimental AP models. In L-arginine-induced AP, miR-141 regulates autophagy by targeting HMGB1. It binds to a specific site in the 3′UTR of HMGB1 mRNA, diminishing HMGB1 expression [119]. miR-21 is upregulated during AP, promoting cellular necrosis via suppressing the expression of the tumor suppressor genes such as FASL and PTEN, inducing RIP1/3-mediated necroptosis [120]. Phosphatase and tensin homolog (PTEN) expression is negatively correlated with caerulein-induced AP and is considered a direct target of miR-216a [121]. In AR42J cells treated with TGF-β, miR-216a levels increased, resulting in decreased PTEN expression and activation of the PI3K/Akt signaling pathway [122]. This pathway is crucial for activating intrapancreatic trypsinogen, a key early event in AP that influences its severity. In acinar cells treated with caerulein, elevated levels of miR-148a-3p targeted and downregulated PTEN mRNA expression. The study also expresses. The study also demonstrated that inhibiting miR-148a-3p reduced necrosis [123]. Furthermore, activation of TGF-β signaling leads to an increase in Smad7 expression. Subsequently, Smad7 negatively regulates the TGF-β signaling via binding to the TGF-β type I receptor. An AP study proved that miR-216a is upregulated and reduces Smad7 expression via binding to the 3′ UTR of Smad7 mRNA, inhibiting its translation, resulting in promoting the TGF-β signaling [122,124]. Additionally, it has been demonstrated that during AP, the main store-operated Ca^2+^ entry (SOCE) channels, specifically Trpc3 and Trpc6, are upregulated at the post-transcriptional level in PACs, contributing to elevated intracellular calcium [Ca^2+^]_i_ levels. Administering a miR-26a mimic either locally to the pancreas or systemically in mice targets these channels, thereby reducing both normal calcium oscillations and pathological calcium spikes in PACs [125] (Table 1).

Severe acute pancreatitis is linked to multiple serious complications and has a mortality rate as high as 30% [126]. Organ failure is a key factor in determining the prognosis of AP, with myocardial injury being the leading cause of death in SAP [127]. It has been reported that miR-29a-3p can be transported by extracellular vesicles derived from mesenchymal stem cells to cardiomyocytes, where it targets and downregulates the expression of HMGB1. As a result, the TLR4/Akt signaling pathway was inhibited, diminishing myocardial inflammation and apoptosis during SAP [128]. Excessive inflammatory response in SAP is associated with excessive release of inflammatory cytokines, such as TNF-α, which induces miR-155 overexpression in the intestinal epithelia. miR-155 suppresses RhoA protein synthesis at the post-transcriptional level. Ultimately inhibiting the two major component proteins of the epithelial apical junctional complex, ZO-1 and E-cadherin expression [129]. Furthermore, exosomes have been identified as important carriers of various genetic materials, including miRNAs. Exosomes are very likely to play an important role in the process of extra-pancreatic organ failure in SAP [130]. The most common type of organ dysfunction in SAP patients is acute lung injury (ALI)-induced respiratory failure [131]. Yang et al. have reported that emodin exerts its therapeutic benefits for ALI associated with SAP via increasing the NOVEL-rno-miR-29a-3p expression in exosomes derived from bronchoalveolar lavage fluid, showing anti-inflammatory effects and protecting against lung injury [132]. Moreover, bone marrow mesenchymal stem cells (BMSCs) were modified to overexpress miR-141 and then administered to animals with CAE-induced SAP. The miR-141-modified BMSCs inhibit the PI3K/mTOR signaling pathway through suppressing the phosphorylation of PI3K and mTOR, inhibiting autophagy in SAP [133] (Table 1). 

Pancreatic stellate cells play a key role in CP development when activated, contributing to fibrosis [134]. Regulated inflammation might result in PSCs’ death or a return to a quiescent state, whereas chronic damage causes ongoing activation and fibrosis. miR-15b and miR-16 control the anti-apoptotic protein Bcl-2. Activated PSCs with miRNA mimic transfection have a lower level of Bcl-2 protein. When Bcl-2 is downregulated, Caspase-3, Caspase-8, and Caspase-9 are increased, which triggers apoptosis. These results indicated that miR-15b and miR-16 can increase PSC apoptosis and decrease fibrosis during CP [135]. In experimental CP, miR-21 is significantly upregulated in activated PSCs, driving the expression of connective tissue growth factor (CCN2). This leads to increased collagen production and fibrosis through a positive feedback loop where miR-21 enhances CCN2 expression, boosting miR-21 levels. Additionally, activated PSCs release exosomes containing CCN2 mRNA and miR-21, which are taken up by other PSCs, promoting further fibrosis. This exosomal transport protects these molecules from degradation and facilitates their spread, contributing to the progressive fibrotic response [136] (Table 1).

**Table 1:** Summary of microRNA in pancreatitis.

MicroRNA	MicroRNAs Status of the Disease	Target Protein	Studied Model	Function of MicroRNAs	Ref.
miR-22	Upregulated	↓ ErbB3	In vitro and in vivo rat model	Promotes apoptosis of PACs	[118]
miR-135a	Upregulated	↓ Ptk2	In vitro and in vivo rat model	Promotes apoptosis of PACs	[118]
miR-141	Given to the mice	↓ HMGB1	In vivo mouse model	Block the process of autophagosome formation.	[119]
miR-19b	Upregulated	NA	In vitro and in vivo rat model	promotes the necrosis of PACs	[137]
miR-21	upregulated	↓ PTEN/FASL	In vivo mouse model	Enhances RIP1/3-mediated necroptosis	[120]
miR-148a-3p	upregulated	↓ PTEN	In vitro and in vivo mouse model	Induces necrosis and inflammatory infiltration	[123]
miR-216a	upregulated	↓ Smad7	In vitro and in vivo mouse model	Activation of TGF-β signaling	[122]
miR-26a	Downregulated	↓ Trpc3 and Trpc6	In vitro, in vivo mouse model, and human data	increases SOCE channel expression and [Ca^2+^]_i_ overload,	[125]
miR-29a-3p	Downregulated	↓ HMGB1	In vitro and in vivo rat model	Inhibits cardiomyocyte apoptosis and inflammation during SAP.	[128]
rno-miR-29a-3p	Downregulated	NA	In vivo rat model	Possesses anti-inflammatory properties and mitigates SAP-ALI.	[132]
miR-141	Given to the rats	↓ PI3K/Mtor	In vivo rat model	inhibits autophagy, and ameliorates the development of SAP	[133]
miR-155	upregulated	↓ RhoA	In vivo mouse model	Inhibits the synthesis of ZO-1 and E-cadherin and disrupts the intestinal epithelial barrier in experimental SAP	[129]
miR-15b and miR-16	Downregulated	↓ Bcl-2	In vitro model	Induce apoptosis of rat PSCs	[135]
miR-21	Upregulated	↑ CCN2	In vitro model	Amplifies fibrotic signaling in the pancreas during CP	[136]

A study examined the expression profiles of miRNAs and mRNAs in patients at risk for CP to develop a risk miRNA–mRNA pathway network and uncover potential molecular mechanisms behind CP. The study demonstrated that hsa-miR-324-5p targets COX5A and, as a result, regulates the oxidative phosphorylation, affecting the ubiquitin-mediated proteolysis through downregulating ANAPC13 [138]. Additionally, proinflammatory cytokines such as TNF-α, IL-1β, and IL-6 are negatively regulated by miR-146a. MiR-146a is downregulated in CP patients, which raises levels of proinflammatory cytokines and chemokines and accelerates the course of the disease [139] (Table 1).

## 5. Phytochemicals for Acute and Severe Acute Pancreatitis

### 5.1. Phytochemicals Target Calcium Signaling

Disturbances in calcium homeostasis represent one of the earliest and most critical initiating events in pancreatitis. Sustained elevation of cytosolic Ca^2+^ leads to premature activation of trypsinogen, mitochondrial dysfunction, and acinar cell necrosis. Phytochemicals such as caffeine, berberine, resveratrol, and curcumin were shown to play a pivotal role in restoring calcium balance by targeting multiple regulatory sites. Caffeine and berberine inhibit IP3R and RyR channels, preventing Ca^2+^ overload, while resveratrol improves mitochondrial Ca^2+^ uptake and protects against ATP depletion [140,141]. Curcumin and urolithin A enhanced Ca^2+^-ATPase activity, preserving the structural integrity of the endoplasmic reticulum and mitochondria [5,59,142]. These actions collectively prevented excessive activation of calcium-dependent proteases and NF-κB signaling, limiting inflammatory and oxidative damage. By maintaining physiological Ca^2+^ oscillations and preventing pathological Ca^2+^ spikes, phytochemicals contributed to both local acinar cell protection and systemic stabilization. Their integrated effect highlights calcium signaling as a key therapeutic target for modulating the severity of both acute and severe pancreatitis. The detailed mechanistic and experimental evidence for these compounds is summarized in Table 2 and Table 3.

#### Phytochemicals Target Calcium Signaling in Other Diseases

Several phytochemicals have been shown to modulate calcium signaling in various diseases. Recently, in ischemia/reperfusion-induced myocardial injury, berberine acts as a potent inhibitor of the mitochondrial calcium uniporter, blocking mitochondrial Ca^2+^ influx [143]. Epigallocatechin-3-gallate, the major catechin found in green tea, protects against liver fibrosis in mice by suppressing IP_3_-mediated calcium signaling, lowering intracellular Ca^2+^, and inhibiting hepatic stellate cell activation [144]. Curcumin acts as an immunosuppressant by inhibiting T-cell activation through blocking extracellular Ca^2+^ entry via CRAC and L-type Ca^2+^ channels, thereby inhibiting Ca^2+^-dependent NFAT activation [142]. Additionally, curcumin and resveratrol alleviate hypoxia-induced neuronal injury by inhibiting TRPM2-mediated Ca^2+^ influx, which reduces mitochondrial Ca^2+^ overload, ROS production, and apoptosis [145,146]. Recently, quercetin protected H9c2 cells from hypertonic stress by preventing cytosolic Ca^2+^ elevation and mitochondrial Ca^2+^ uptake, thus blocking MPTP opening and apoptosis [147,148]. Baicalin suppresses TRPV1 upregulation in dorsal root ganglion neurons, decreasing Ca^2+^ influx and alleviating neuropathic pain [149]. Ginsenoside Re exhibits neuroprotective effects in an excitotoxicity model by attenuating L-type Ca^2+^ channel-mediated calcium overload and mitochondrial dysfunction [150].

### 5.2. Phytochemicals Target Ferroptosis

Ferroptosis, an iron-dependent form of regulated cell death, contributes significantly to the progression of pancreatitis by driving lipid peroxidation, mitochondrial injury, and acinar cell death [63]. Several phytochemicals have been identified to counteract ferroptotic injury through antioxidant reinforcement and modulation of iron metabolism. Wedelolactone, matrine, and neferine suppress lipid ROS generation and upregulate GPX4, a key enzyme that neutralizes lipid peroxides [72,151,152]. Resveratrol and curcumin were shown to reduce ACSL4 expression and inhibit ferroptotic sensitivity, while morin hydrate prevents iron accumulation and improves mitochondrial function. Through these concerted actions, phytochemicals prevent ferroptotic cell death and sustain antioxidant defenses, leading to decreased inflammatory injury and improved tissue recovery. In severe pancreatitis, their modulation of ferroptosis correlates with improved organ function and reduced systemic complications. The ferroptosis-targeting phytochemicals, their experimental models, and mechanistic pathways are summarized in Table 2 and Table 3.

### 5.3. Phytochemicals Target NF-κB

NF-κB signaling is a central mediator of inflammation in pancreatitis, orchestrating the transcription of cytokines, chemokines, and adhesion molecules [153]. Phytochemicals such as curcumin, luteolin, berberine, and apigenin act at multiple points along this pathway. These compounds suppress IκB kinase activation, preventing degradation of IκB and subsequent nuclear translocation of the NF-κB p65 subunit [154]. This inhibition results in decreased expression of TNF-α, IL-1β, IL-6, and MCP-1, ultimately reducing inflammatory infiltration and acinar necrosis [155].

Furthermore, phytochemicals like berberine and curcumin show cross-regulation between NF-κB and MAPK pathways, collectively reducing oxidative stress and fibrogenic signaling. In severe pancreatitis models, their sustained suppression of NF-κB signaling mitigates systemic inflammatory response syndrome (SIRS) and improves survival outcomes. Table 2, Table 3 and Table 4 summarize these NF-κB inhibitory phytochemicals and their specific molecular targets.

### 5.4. Phytochemicals Targeted the p38-MAPK Pathway

The p38 MAPK pathway acts as a stress-responsive kinase cascade, influencing cytokine production, apoptosis, and oxidative injury [107,156]. In pancreatitis, hyperactivation of p38 MAPK exacerbates inflammation and cell death [157]. Phytochemicals, including galangin, suppressed p38 phosphorylation and its downstream targets, such as MK2 and HSP27, leading to reduced inflammatory cytokine expression [86,158].

In addition, baicalin demonstrated dual inhibition of p38 and JNK activation, resulting in attenuated oxidative damage and apoptotic signaling [159]. In severe pancreatitis, phytochemicals targeting p38 MAPK help restore redox balance, minimize tissue injury, and prevent multiple organ dysfunction. Their actions often complement NF-κB inhibition, forming a coordinated anti-inflammatory network. The associated compounds and their experimental validations are summarized in Table 2, Table 3 and Table 4.

### 5.5. Phytochemicals Target the Nrf2 Pathway

NRF2 functions as a master regulator of antioxidant and cytoprotective gene expression, serving as a key adaptive mechanism against oxidative stress [77,78]. Phytochemicals such as dihydrokaempferol and visnagin, and many more, were shown to activate the NRF2/HO-1 signaling axis by disrupting the Keap1–NRF2 complex, leading to nuclear translocation of NRF2 and transcription of downstream antioxidant genes, including SOD, CAT, GPx, and NQO1 [27,160]. These antioxidant responses limit ROS accumulation and lipid peroxidation while preserving mitochondrial function [161]. Curcumin and thymoquinone enhanced this response further by promoting heme oxygenase-1 expression and improving glutathione turnover [162,163]. Collectively, activation of the NRF2 pathway by phytochemicals offers a potent defense against oxidative stress and systemic complications of pancreatitis. Table 2, Table 3 and Table 4 provide a summary of NRF2-activating compounds and their documented protective mechanisms.

### 5.6. Phytochemicals Target MicroRNAs

MicroRNAs (miRNAs) are emerging post-transcriptional regulators of inflammation, apoptosis, and fibrosis in pancreatitis [117,125,137,139]. Phytochemicals such as emodin, berberine, and quercetin influence miRNA expression profiles, thereby modulating critical molecular pathways [132,164]. Curcumin was shown to upregulate miR-29a to inhibit fibrogenic signaling through TGF-β suppression [165], while berberine and resveratrol downregulated miR-21 and miR-155, reducing NF-κB-driven cytokine release [166]. These interactions extend beyond local pancreatic protection to systemic regulation, with phytochemical-modulated miRNAs influencing autophagy, apoptosis, and epithelial barrier integrity. In severe pancreatitis, modulation of circulating and exosomal miRNAs by these compounds presents a novel therapeutic avenue. All miRNAs and their phytochemical modulators are summarized in Table 1.

### 5.7. Phytochemicals Target Other Pathways

#### 5.7.1. Mitochondrial Dysfunction

Mitochondrial dysfunction is a pivotal event in pancreatitis, resulting in impaired ATP generation, excessive ROS production, and loss of mitochondrial membrane potential (ΔΨm), which together drive acinar cell necrosis and amplify inflammation [167,168]. Targeting mitochondrial stability with phytochemicals offers significant protection [169]. Dihydrodiosgenin (Dydio) prevents mitochondrial depolarization, reduces ROS accumulation, preserves ATP levels, and alleviates pancreatic injury through inhibition of the PI3K/Akt pathway [170]. Isorhamnetin restores mitochondrial function and reduces acinar damage by modulating the KDM5B/HtrA2 signaling axis [171]. Similarly, Rhizoma Alismatis Decoction (RAD) enhances mitochondrial autophagy and promotes the clearance of damaged organelles by upregulating LC3-II, Beclin-1, and ATG5 while downregulating p62, thereby improving cellular homeostasis and reducing apoptosis [172]. Collectively, these phytochemicals mitigate mitochondrial dysfunction and restore energy metabolism, thereby protecting acinar cells and limiting pancreatic injury. The detailed experimental models, doses, and mechanistic outcomes of these phytochemicals are summarized in Table 2, Table 3 and Table 4.

#### 5.7.2. Oxidative Stress and Inflammation

Oxidative stress and inflammation are intertwined drivers of pancreatic damage. Polyphenolic phytochemicals such as curcumin, luteolin, and epigallocatechin gallate (EGCG) exhibited dual antioxidant and anti-inflammatory effects by scavenging reactive oxygen species and inhibiting enzymes such as COX-2, MPO, and iNOS [173,174]. These effects reduce edema, neutrophil infiltration, and necrosis. By restoring antioxidant enzyme levels and inhibiting proinflammatory mediators, phytochemicals interrupt the ROS–cytokine feedback loop that propagates tissue damage. Combined with calcium and MAPK modulation, their actions yield a comprehensive anti-inflammatory effect [174,175,176]. Table 4, Table 5 and Table 6 summarize the key antioxidant and anti-inflammatory phytochemicals and their experimentally validated actions.

**Table 2:** Summary of phytochemicals in acute pancreatitis (↑ increased, ↓ decreased).

Phytochemical Name	Class/Plant	Therapeutic Dose/Route of Administration	Main Target Pathway/Findings	Ref.
			Calcium signaling	
Berberine	Isoquinoline alkaloid *Coptis chinensis*, *Phellodendron chinensis*, or three needles	10 µM in vitro	↓ M3 muscarinic receptor, Ach-induced Ca^2+^ oscillations	[141]
Caffeine	Methylxanthine alkaloid *Coffea arabica Camellia sinensis*	1, 5, 10, or 25 mg/kg i.p.	↓ IP_3_R activity, Cytosolic Ca^2+^ overload	[140]
Emodin	Anthraquinone rhubarb	10 and 20 μM In vitro	↓ Ca^2+^ concentration, Bip, PERK, ATF6, IRE1	[177]
Chaiqinchengqi decoction	Genus: *Artemisiae Scopariae*, *Gardenia,* and *Rhubarb*	10 mL/kg Oral	↑ SERCA2, ER Ca^2+^ reuptake, ↓ Cytosolic Ca^2+^ overload, acinar cell necrosis	[178]
Mogroside IIE	Triterpenoid saponin Unripe *Siraitia grosvenorii*	10 mg/kg i.p.	↓ IL-9, IL-9R signaling, Cytosolic Ca^2+^ overload, LC3-II, Cathepsin B activity, ↑ p62	[179]
			Ferroptosis pathway	
Silibinin	Flavonoid milk thistle (*Silybum marianum*)	100 mg/kg i.p.	↓ FAT10, NCOA4, TFRC, ACSL4, ↓ Free Fe^2+^ ↑ FTH1	[70]
Wedelolactone	Coumarin-like compound *Eclipta prostrata*	25 mg/kg or 50 mg/kg i.p.	↑ GPX4 → ↓ Ferroptosis	[151]
			NF-κB pathway	
8α-Hydroxypinoresinol	Lignan *Nardostachys jatamansi*	0.5, 5 or 10 mg/kg i.p.	↓ IκBαdegradation, NF-κB p65, TNF-α, IL-1β, IL-6	[50]
Artemisinin	Sesquiterpene lactone *Artemisia annua*	50 mg/kg i.p.	↓ NF-κB, MIP-1α, IL-1β ↑ Caspase-3	[180]
Betulinic acid	Pentacyclic Triterpenoid *Betula platyphylla*	1, 5, or 10 mg/kg i.p	↓ IκBα degradation, NF-κB p65, TNF-α, IL-1β, IL-6, CCL2, CXCL2, MPO	[181]
Genistein	Flavonoids soy and other legumes	10, 100 mg/kg i.p	↓ NF-κB p65, TNF-α, iNOS, COX-2, MPO	[182]
Myricetin	Flavonoid fruits and vegetables	0.5, 2, 5 mg/kg i.p.	↓ Calcineurin activity, CaMKK2, CaMKIV, NF-κB, TNF-α, IL-6, IL-1β, Cathepsin B ↑ AMPK, SIRT1	[94]
Nanocurcumin	Polyphenol *Curcuma longa*	100 mg/kg/day	↓ TLR4, NF-κB p65, TNF-α	[98]
Nimbolide	Limonoid (from *Azadirachta indica*/neem)	0.3 and 1 mg/kg i.p	↓ IκBα degradation, NF-κB p65, TNF-α, IL-1β, IL-6, iNOS, nitrotyrosine, ↑ SIRT1	[183]
Protocatechuic acid	Phenolic acid *Ginkgo biloba,* *Hibiscus sabdariffa* L.	50, 100 mg/kg oral	↓ HMGB1, TLR4, NF-κB p65, IL-1β, IL-6, TNF-α	[184]
Rosmarinic acid	Polyphenolic caffeic acid ester rosemary, sage, lemon balm	50 mg/kg i.p.	↓ NF-κB p65, TNF-α, IL-1β, IL-6	[185]
Tricetin	Flavonoids *Eucalyptus*	10, 30 mg/kg i.p.	↓ NF-κB p65, MPO, TNF-α, IL-6, PARP1	[186]
			MAPK pathway	
Anemarsaponin B	Steroidal saponin *Anemarrhena asphodeloides*	20, 40, 80 mg/kg i.p.	↓ Occludin-TAK1, p-JNK, p-ERK, p-p38, TRAF6, TNF-α, IL-1β, IL-6, cleaved caspase-3 ↑ SOD, GSH-Px	[109]
Calycosin	Isoflavonoid *Radix astragali*	25, 50 mg/kg i.p.	↓p38 MAPK and NF-κB, NF-α, IL-6, IL-1β, MDA ↑ SOD, GSH-Px	[187]
Curcumin	Polyphenol *Curcuma longa*	200 mg/kg i.p.	↓ Phosphorylation of p38 MAPK, TNF-α, and CRP	[173]
Dihydromyricetin	Flavonoid *Ampelopsis grossedentata*	25, 100 mg/kg i.p.	↓ TRAF3, TRAF3–MKK3, p38 MAPK, IL-1β, TNF-α, IL-17	[188]
Glycyrrhizin	Triterpenoid saponin *Glycyrrhiza glabra*	NA	↓ ERK^1/2^, STAT3, AKT	[189]
Green tea polyphenols	Catechins *Camellia sinensis*	25 mg/kg i.p.	↓ NF-κB, NF-κB p65, TNF-α, TGF-ββ, VEGF, ICAM-1, P-selectin, PARS	[190]
Ligustrazine	Alkaloid *Ligusticum wallichii*	150 mg/kg day i.p.	↓ p38 MAPK, ERK1/2, TNF-α, IL-1β, IL-6 ↑ p53, cleaved caspase-3	[191]
Stigmasterol	Phytosterol, vegetable oils, nuts, seeds, and legumes	50, 100 mg/kg i.p.	↓ ERK^1^, KRAS, B-RAF, TNF-α, IL-6, IL-1β	[192]
			Nrf2 pathway	
Galangin	Flavonol *Plantago major* L., *Alpinia officinarum Hance*, and *Scutellaria galericulata* L.	50 mg/kg Oral	↓ ROS levels, M1 macrophage polarization ↑ Nrf2, SRXN1	[158]
Tanshinone IIA	Diterpenoid quinone *Salvia miltiorrhiza*	5, 25, 50 mg/kg i.p.	↓ ROS, MDA ↑ Nrf2, ↑ HO-1	[79]
Triptolide	*Diterpene triepoxide Tripterygium wilfordii* Hook.f.	50, 100 μg/kg Oral	↑ Nrf2, HO-1, SOD1, GPx1, NQO1, GSH, SOD ↓ ROS and MDA, NF-κB p65	[193]
Visengin	Furanocoumarin derivative *Ammi visnaga*	10, 30, 60 mg/kg oral	↑ Nrf2, HO-1, NQO1 ↓ NF-κB, TNF-α, IL-6, IL-1β	[27]
			Antioxidant/Anti-inflammatory pathway	
Chlorogenic acid	Polyphenols, coffee beans, cocoa leaves and seeds, yerba mate	20 mg/kg oral	↓ MPO, MIF, Serum amylase	[194]
Withaferin A	Steroidal lactone roots of *Withania somnifera*	2 and 10 mg/kg Oral	↓ MDA, NO, MPO, nitro tyrosine ↑ GSH	[195]
			Mitochondrial dysfunction	
Dihydro- diosgenin	Steroidal saponins *Dioscorea zingiberensis* C. H.	(5 or 10 mg·kg^−1^) i.p.	↓ (ΔΨm) loss, ATP depletion, ROS, PI3K/Akt pathway	[170]
Dioscorea zingiberensis	Phenolic compounds rhizomes of *D. zingiberensis*	0.5 mM of compound 6 In vitro	↓ATP depletion and ROS generation	[196]
Rhizoma Alismatis Decoction	Genus: *Alisama,* Juzep *Atractylodes, macrocephala* Koidz	(36 g crude drug/kg/d) 4 g crude drug/kg Oral	↓ p16INK4a, p21, p62 ↑ Beclin-1, ATG5, LC3-II	[172]
			MicroRNA pathway	
Baicalin	Flavonoid *Scutellaria baicalensis* Georgi	50, 100 mg/kg (i.p.) (5−75 μM) in vitro	↑ miR-15a, ↓ MAP2K4, CDC42, MAP3K1 ↓ miR-136-5p/↑ SOD	[159,197]
Quercetin	Flavonoid Fruits and vegetables	50 and 100 mg/kg oral	↑ miR-216b ↓ MAP2K6, NEAT1, TRAF2	[17]
Pinocembrin	Flavonoid propolis	10 mg/kg oral	↓ miR-34a-5p → ↑ SIRT1 → ↑ PPAR-α and IκB-α → ↓ NF-Κb/↑ Pancreatic Nrf2 and HO-1	[16]

**Table 3:** Summary of phytochemicals in severe acute pancreatitis.

Phytochemical Name	Class/Plant	Therapeutic Dose/Route of Administration	Main Target Pathway/Findings	Ref.
			Calcium signaling	
Resveratrol	Polyphenol Grape	10 mg/kg i.v	↑ Ca^2+^-ATPase activities, Ca^2+^-Mg^2+^-ATPase activity, ↓ [Ca^2+^]i, PLA2activity	[198]
Urolithin A	Coumarins Pomegranate (*Punica granatum*)	30 mg/kg i.p.	↓ IP_3_R, VDAC1, GRP75, MPTP opening, Cytochrome c release, RIPK1/RIPK3	[59]
			Ferroptosis pathway	
Da Cheng Qi Decoction	Decoction of rhubarb, mirabilitum, bitter orange, and magnolia bark	7 g/kg oral	↓ Ferroptosis ↓ NOX2, ROS ↑ GPX4	[199]
Matrine	Alkaloid herbal plants	200 mg/kg i.p.	↓ Ferroptosis ↑ UCP2, PGC1α, GPX4, SLC7A11	[72]
Neferine	Bisbenzylisoquinoline alkaloids of *Nelumbinis plumula*	50, 75 mg/kg i.p.	↓ Ferroptosis ↑ NRF2, HO-1, GPX4, FPN	[152]
			NF-κB pathway	
Berberine	Isoquinoline alkaloid *coptis chinensis, phellodendron chinensis*, or three needles	10 mg/kg i.p.	↓ NF-κB p65, JNK, TNF-α, IL-1β, IL-6, MPO	[200]
Colchicine	Alkaloid *Autumn crocus*	0.5 mg/kg oral	↓ NF-κB p65, TNF-α, IL-1β, IL-6, STAT3, AKT, iNOS, MPO, ROS	[201]
Isoacteoside	Coumaricacids *Monochasma savatieri* Franch. Ex Maxim	40 mg/kg i.p.	↓ TLR4, NF-κB p65, TNF-α, IL-6, MPO, NO	[202]
			p38/MAPK pathway	
Albiflorin	Monoterpenoid *Paeonia lactiflora* Pall. or *Paeonia veitchii* Lynch	5, 10, and 20 mg/kg i.p.	↓ p-p38 MAPK, NF-κB p65, ALT, AST, TNF-α, IL-6, and IL-1β, MDA ↑ SOD and GSH-Px	[203]
Quercetin	Flavonoid Fruits and vegetables	50 mg/kg i.p.	↓ TLR4, MyD88, p-p38 MAPK, IL-1β, TNF-α, IL-6, IL-17, Bip, p-IRE1α, sXBP1, p-eIF2α, ATF6 ↑ ZO-1, occludin, claudin-1	[204]
			Nrf2 pathway	
Dihydro- kaempferol	Flavonoid *Bauhinia championii* (Benth)	20, 40, 80 mg/kg oral	↓ Keap1, ↓ MDA, and ROS ↑ Nrf2, HO-1, NQO1, GSH	[160]
Galangin	Flavonol lesser galangal	10, 20, or 40 mg/kg Oral	↑ Nrf2, HO-1 ↓ TNF-α, IL-1β, ROS	[86]
Kaempferol	Flavonoid leafy green vegetables: broccoli, cabbage, spinach,	25 or 50 mg/kg KA (oral); 2.5 or 5 mg/kg (DTM@KA NPs) i.v	↑ Nrf2, GSH, Drp1, Pink1/Parkin, ATP production ↓ ROS	[205]
Micheliolide	Sesquiterpene lactone *Michelia champaca*	25, 50 mg/kg Oral	↑ Nrf2, HO-1, NQO1 ↓ NF-κB p65, TNF-α, IL-1β, ROS	[89]
			Mitochondrial dysfunction	
Isorhamnetin	methylated derivative of quercetin	10, 30 mg/kg i.p.	Mitochondrial dysfunction ↓ ROS, mtDNA, KDM5B ↑ ATP, HtrA2	[171]
			MicroRNA	
Emodin	Anthraquinone *Rheum palmatum* L., *Polygonum multiflorum* Thunb., and *Polygonum cuspidatum* Siebold and Zucc.	40 mg/kg Oral	↑ rno-miR-29a-3p ↓ Macrophage activation	[132]
Salidroside	phenolic glycoside *Rhodiola rosea* L.	20 mg/kg i.p.	↓ miR-217-5p, p38 MAPK ↑ YAF2	[206]

**Table 4:** Summary of phytochemicals in chronic pancreatitis. (↑—increased; ↓—decreased).

Phytochemical Name	Class/Plant	Therapeutic Dose/Route of Administration	Main Target Pathway/Findings	Ref.
Puerarin	Isoflavones Radix Puerariae	100 mg/kg Oral	MAPK signaling ↓ JNK1/2, ERK1/2,p38 MAPK, α-SMA, Fibronectin, Col1α1, GFAP	[115]
Curcumin	Polyphenol *Curcuma longa*	10 or 20 mg/kg i.p.	Nrf-2 pathway ↑ Nrf2, HO-1 ↓ TGF-β1, α-SMA, Col1a1, Col4a1, Fn1	[26]
Catechin hydrate	Flavonoids green tea	1, 5 or 10 mg/kg i.p.	TGF-β/Smad2 ↓ TGF-β1, Smad2, α-SMA/Acta2, fibronectin 1, collagen-I/III/IV	[207]
*Psidium guajava* Flavonoids	Flavonoids *Psidium guajava*		Anti-inflammatory ↓ NLRP3, IL-1β, IL-18, Caspase-1	[208]

**Table 5:** Clinical trials with phytochemicals used as a potential treatment for pancreatitis.

Phytochemical/Intervention	Study Type and Setting	Population (*n*)	Type of Model/Control Used	Dose/Route/Duration	Key Outcomes	Statistical Significance	Safety/Toxicity	Major Limitations	Ref.
Chinese herbal medicine + Western medicine (broader AP population)	Systematic review and meta-analysis (Front Pharmacol. 2025)	Multiple RCTs pooled (counts vary by endpoint)	Clinical population (acute pancreatitis); controls = standard Western care	Varies; oral decoctions most common	↑ clinical efficacy (RR≈1.26); ↓ TNF-α, IL-6; ↓ time to pain relief; ↓ ICU stay	All pooled endpoints statistically significant (*p* < 0.05)	Safety acceptable overall; AE reporting heterogeneous	Risk of bias; heterogeneity in interventions and endpoints	[209]
Nanocurcumin (adjuvant to standard care)	Double-blind RCT; single center (Iran)	Mild/moderate AP (*n* = 42; 21 vs. 21)	Randomized control; placebo group (identical capsule)	40 mg nanocurcumin twice daily (oral), 2 weeks	↓ GI ward length of stay; ↓ analgesic requirement; ↑ appetite score	*p* < 0.05 for all endpoints	No adverse effects reported; no mortality; no withdrawals	Small sample; single center; short follow-up; adjuvant use only	[210]
Chinese herbal medicine (various multi-herb formulas) + Western medicine vs. Western medicine alone	Systematic review and meta-analysis of RCTs in hyperlipidemic AP	50 trials; *n* = 3635 total	Clinical trials vs. Western medicine control arms	Varies by formula; mostly oral decoctions; inpatient courses	Improved composite clinical efficacy; ↓ inflammatory markers; faster symptom resolution	Pooled effect sizes significant (*p* < 0.01)	Generally reported as safe; adverse event reporting inconsistent/limited	Heterogeneity; variable formula composition; risk of bias concerns	[211]
Dachengqi (Chaiqinchengqi) series formulas (TCM)	Randomized trials and meta-analyses in AP/SAP (China)	Multiple small-to-moderate RCTs	AP/SAP patients vs. standard care; placebo/sham in some	Oral/NG decoctions; duration varies	↓ mortality; ↓ hospital stay; ↓ surgery rate; ↓ serum resistin (some trials)	*p* < 0.05 in most studies; meta-analytic effect confirmed	Generally, well tolerated; variable AE reporting	Methodological quality variable; publication bias possible; formula heterogeneity	[212]
Resveratrol (pre-ERCP to prevent post-ERCP pancreatitis)	Registered clinical trial (status unclear)	Adults undergoing ERCP (planned)	Preventive model; placebo control planned	Oral resveratrol pre-procedure (planned regimen)	Prevention of post-ERCP pancreatitis (primary)	Not reported/trial results pending	Not reported	Results not posted/unclear	[213]

Note: Available clinical data on phytochemicals in pancreatitis are limited. Most evidence arises from small-scale or adjunctive trials; larger, well-designed RCTs with standardized dosing and safety assessments are needed.

**Table 6:** Overview of preclinical animal models of pancreatitis: induction protocols, severity readouts, outcomes, and limitations.

Study/Ref.	Species/Strain and Sex	Control Details	Model (Trigger)	Induction Details (Dose, Route, Schedule)	Severity Determination (Pre-Specified Readouts)	Primary Outcomes	Statistical Significance	Toxicity/Safety	Key Model Limitations
AP	Mouse (C57BL/6J), Male	Saline-injected controls under identical timing and handling	Caerulein (CCK analog)	50 μg/kg IP, hourly × 6 (±LPS 10 μg/kg)	Serum amylase/lipase; pancreatic histology score (edema/infiltrate/vacuolization/necrosis); MDA; cytokines	↓ Enzyme rise; ↓ histologic injury; ↓ MPO	*p* < 0.05 vs. control	No weight loss; no mortality	Mild, reversible edema-predominant AP; limited necrosis
SAP	Mouse (C57BL/6J), Male	Sham-operated mice (saline duct infusion)	NaT retrograde infusion	2.5% NaT, 10 μL/min × 3 min via biliopancreatic duct	% necrosis; hemorrhage; ALI score; lung MPO; BUN/Cr; IL-6	↓ Pancreatic necrosis; ↓ ALI; ↑ ATP	*p* < 0.01 for biochemical and histologic scores	Peri-op mortality reported	Surgical model; uneven injury near the main duct
AP (alcoholic)	Rat, Male	Vehicle (ethanol alone)	FAEE (EtOH + POA)	Ethanol + palmitoleic/oleic acid, IP	Ca^2+^ overload indices; mitochondrial ΔΨm; amylase/lipase; histology	↓ Ca^2+^ overload; ↓ necrosis	*p* < 0.05	–	Protocol/strain variability
CP	Mouse, Male	Saline-injected controls	Repeated caerulein	50 μg/kg/h, 6 h/day, 2 days/week × 10 weeks	Fibrosis score; Sirius Red; hydroxyproline; α-SMA/Col-I	↓ Fibrosis; ↓ PSC activation markers	*p* < 0.001	–	Models fibrosis but not pain/duct obstruction
CP	Rat, Male, Female	Sham laparotomy without duct ligation	PDL	Ligation of pancreatic duct(s)	Atrophy/fibrosis; ECM markers	↓ Fibrosis progression	*p* < 0.05	Surgical risk	Mouse ductal anatomy complicates uniform PDL

## 6. Clinical Evidence of Phytochemical Interventions in Pancreatitis

### 6.1. Nanocurcumin

Curcumin, ((1E,6E)-1,7-bis (4-hydroxy-3-methoxyphenyl)-1,6-heptadiene-3,5-dione) also known as diferuloylmethane, has a long history of use as a traditional medicine since it is nontoxic and has been shown to have antioxidant, analgesic, anti-inflammatory, antiseptic, and anticarcinogenic activity [214]. Nanocurcumin has demonstrated anti-inflammatory effects in preclinical AP by inhibition of the TLR4/NF-κB signaling pathway [98]. A double-blind, parallel-arm randomized controlled trial was conducted with a total of 42 patients with mild-to-moderate AP to evaluate nanocurcuminefficacy and safety. The study showed that it can be safely used as an adjuvant therapy and significantly decreased both gastrointestinal ward length of stay (LOS) and analgesic use, while improving patient appetite during the study period [210].

### 6.2. Chaiqinchengqi Decoction

Chaiqinchengqi decoction is a TCM and consists of multiple herbs, and it has been shown to have antioxidant and anti-inflammatory effects in both in vitro and in vivo studies [215,216,217]. Recently, an AP study has indicated that Chaiqinchengqi shows a regulatory effect on the metabolic trajectory of pancreatic tissue [218]. 248 adults with predicted moderately severe or severe AP participated in a prospective, pragmatic, randomized controlled trial, which aimed to evaluate the Chaiqinchengqi decoction on clinical outcomes in AP patients. The study found that Chaiqinchengqi decoction, when used as an adjunctive therapy, significantly reduced the duration of respiratory failure and enhanced the 6-month clinical outcomes of AP [219].

### 6.3. Dachengqi Decoction

Da Cheng Qi decoction is a well-known Chinese herbal formula, consisting of many herbal ingredients such as rhubarb, mirabilitum, and magnolia bark. It has been evaluated in a clinical study conducted on 40 patients with hypertriglyceridemia AP (HLAP). The study used the modified Da Cheng Qi decoction, consisting of 16 herbs and given in combination with conventional therapy to the intervention group, and the control group received only conventional treatment. The results showed that the combination treatment increased the cure rate by 60% vs. 25% in the control, in addition to a significant decrease in triglycerides, serum amylase, leukocyte count, and neutrophil ratio in the intervention group [220]. In a randomized clinical trial, Jiang et al. (2016) [221] demonstrated that modified Da Chengqi granules can promote immune function in early SAP patients. The sample size was 77 patients, and they were divided into a control group that received only conventional therapy and an experimental group that was treated with the conventional treatment combined with modified DaChengqi granules. Adding these granules to the treatment reduced mortality, serum cytokine levels, immunological markers, and endotoxins in the experimental group significantly compared to control patients, revealing the beneficial role of the granules as adjunctive therapy [221].

## 7. Preclinical Animal Models and Severity Assessment: Models, Readouts, and Limitations

Animal models are still essential for understanding the pathophysiology of pancreatitis and for evaluating preclinical medications [222]. Caerulein (CCK analog) hyperstimulation, ethanol + fatty acid ethyl ester (FAEE) exposure, basic amino acid overload (e.g., L-arginine), retrograde bile-salt (NaT/TCA) infusion, and dietary choline-deficient/ethionine-supplemented (CDE) feeding are the primary experimental methods; pancreatic duct ligation (PDL), and long-term toxin or alcohol co-exposure are used to simulate chronic injury. Caerulein (edematous AP), FAEE (alcoholic AP with mitochondrial Ca^2+^ excess), L-arginine (necrotizing AP), NaT/TCA (hemorrhagic SAP with multiorgan injury), CDE (female-predominant severe AP), and PDL (progression from acute to chronic fibrosis) are the human phenotypes that each model replicates. Elevations of serum amylase/lipase, ALT/AST, BUN/creatinine, composite histology scores for edema, inflammation, and necrosis, lung and renal injury indices (wet/dry ratio, MPO, IL-6, TNF-α), tissue oxidative stress markers (MDA, ROS, mitochondrial membrane potential, ATP), and, in chronic models, fibrosis scoring (Masson’s trichrome/Sirius Red, hydroxyproline, α-SMA/Collagen-I). These predetermined endpoints were used to establish severity groups (mild, severe, and necrotizing). Surgical variability, limited chronic pain reproduction, species/strain and sex variations (e.g., CDE is primarily effective in females), and the absence of comorbidities like obesity or alcoholism are some of the model’s shortcomings. Each animal trial was presented using the same methodology utilized for clinical data in order to increase translational value: (i) model and dose; (ii) route and timing; (iii) primary outcomes; (iv) severity definition; and (v) safety/toxicity measurements.

## 8. Assessment of Lipinski’s Rule of Five and Absorption Properties

Absorption is an essential pharmacokinetic characteristic that influences the efficiency with which a drug enters the bloodstream from its site of administration (e.g., oral, dermal). Inadequate absorption may result in suboptimal drug concentrations, thereby diminishing therapeutic effectiveness. Factors that influence absorption encompass solubility, permeability, and molecular characteristics. pKCSM predicts absorption by utilizing parameters such as Caco-2 permeability, human intestinal absorption (HIA), and P-glycoprotein interactions. It employs graph-based signatures to represent small-molecule chemistry and topology, facilitating the prediction of Lipinski and pharmacokinetic parameters. The smaller size of the drug molecule leads to a decreased contact surface with the target, thereby reducing its binding affinity [223].

A compound’s appropriateness for oral administration is determined by intestinal absorption, Caco-2 permeability, Lipinski’s Rule of Five (Ro5), and interactions with P-glycoprotein (Pgp). Lipinski’s Rule of Five is considered a key principle for forecasting oral bioavailability [224]. Good absorption, distribution, and permeability are more likely to be seen in compounds that adhere to Ro5 (molecular weight ≤ 500 g/mol, ≤5 hydrogen bond donors, ≤10 hydrogen bond acceptors, and logP ≤ 5). Smaller compounds, such as resveratrol and visnagin, conform to Ro5, indicating good oral bioavailability. Larger substances like glycyrrhizin and galanin-like peptide, on the other hand, surpass these thresholds, suggesting possible absorption issues and calling for different delivery strategies.

One important factor influencing oral bioavailability is intestinal absorption; greater absorption is indicated by larger percentages (>80%). Strong choices for oral treatment include compounds with exceptional absorption, such as betulinic acid (99.76%) and caffeine (99.27%). On the other hand, mogroside IIE (11.46%) and baicalin (26.22%) show poor absorption, perhaps because of their bulky or polar structures, which may restrict their therapeutic potential unless they are altered or administered non-orally. The capacity of a chemical to pass through intestinal epithelial cells is measured by Caco-2 permeability; greater results (>0.9 × 10^−6^ cm/s) indicate better permeability. The high permeability of compounds like tanshinone IIA (1.419) and berberine (1.734) supports their oral bioavailability. Curcumin (−0.093) and chlorogenic acid (−0.84), on the other hand, have limited permeability, which may prevent their absorption because of their polarity or contact with efflux transporters. P-glycoprotein interactions have a major impact on medication bioavailability. By pumping substrates out of cells, the efflux transporter Pgp can lower intracellular drug concentrations. Pgp substrates, such as quercetin, baicalin, and curcumin, may restrict their absorption unless they are taken alongside Pgp inhibitors. It is interesting to note that certain phytochemicals, such as silibinin and withaferin A, function as Pgp inhibitors, which may improve the absorption of associated medications or of the phytochemicals themselves by obstructing efflux pathways (Table 7).

## 9. Discussion

Pancreatitis can be broadly classified into three types: acute pancreatitis (AP), severe acute pancreatitis (SAP), and chronic pancreatitis (CP). Among these, AP is the most extensively studied experimental model, primarily due to its high prevalence, reproducibility, and ease of induction. Consequently, AP models have provided substantial mechanistic insights into acinar cell injury and death, uncovering novel pathways, including the role of SARAF and SOCE-mediated Ca^2+^ dysregulation, ferroptosis, and miRNA regulation.

The most commonly employed method for AP induction is cerulein (CAE) administration, which generates a reproducible, reliable, and cost-effective model [28]. However, CAE-induced pancreatitis primarily mimics the mild edematous form observed in humans. To replicate severe disease features, alternative approaches such as the choline-deficient ethionine-supplemented (CDE) diet are utilized [35]. For CP, repeated CAE injections over several weeks are commonly applied, initially causing acute, reversible pancreatic injury that eventually progresses to fibrosis and chronic disease. This approach remains widely used due to its simplicity and reproducibility [39]. SAP models have also been developed to capture systemic complications. In mice, a “two-hit” L-arginine protocol induces pancreatic acinar cell necrosis accompanied by extra-pancreatic manifestations, including acute lung and kidney injury as well as increased intestinal permeability [36]. Another well-established SAP model employs sodium taurocholate (NaT) infusion in C57BL/6 mice, which are considered the gold standard for studying SAP pathophysiology. This model recapitulates several key clinical features of human biliary pancreatitis [37].

The pathogenesis of pancreatitis is multifactorial, involving numerous interdependent molecular pathways that drive disease initiation and progression. Although the three major forms of pancreatitis share common mechanistic pathways, they differ in severity and clinical course. Acute pancreatitis (AP) is initiated by intracellular calcium overload, which promotes premature zymogen activation, mitochondrial dysfunction, and excessive production of reactive oxygen species (ROS), ultimately leading to local inflammatory responses in pancreatic acinar cells (PACs) [32]. Damaged PACs subsequently recruit immune cells, which release cytokines and chemokines while upregulating adhesion molecules, thereby attracting neutrophils, monocytes, and macrophages. The interaction between necrotic PACs and infiltrating immune cells amplifies both local and systemic inflammation [225], which in severe cases contributes to multiorgan dysfunction (MOD), a hallmark of severe acute pancreatitis (SAP). Recurrent episodes of AP may further progress to chronic pancreatitis (CP), characterized by irreversible pancreatic damage and fibrosis, ultimately resulting in both exocrine and endocrine insufficiency [4].

Calcium plays a central role in the pathogenesis of pancreatitis. Intracellular Ca^2+^ overload arises from multiple dysregulated mechanisms, including impaired activity of Ca^2+^-extruding proteins such as the plasma membrane Ca^2+^ ATPase (PMCA) [226], downregulation of SARAF expression [50], excessive release of Ca^2+^ from intracellular stores such as the endoplasmic reticulum (ER) via ryanodine receptors (RyR) and inositol 1,4,5-trisphosphate receptors (IP_3_R), and activation of store-operated calcium entry (SOCE) following ER depletion. Sustained calcium overload initiates a cascade of cell death and inflammatory signaling, including activation of NFAT and NF-κB pathways [8,9,53], mitochondrial dysfunction, reactive oxygen species (ROS) generation, and premature trypsinogen activation. In parallel, MAPK signaling is triggered, driving the transcription of proinflammatory genes and further amplifying pancreatic injury. All of these events lead to inflammation, apoptosis, and ultimately the death of PAC. Beyond necrosis and apoptosis, ferroptosis has recently been identified as a distinct, iron-dependent form of regulated cell death. Emerging evidence implicates ferroptosis in the pathophysiology of both acute pancreatitis (AP) and severe acute pancreatitis (SAP). The cytoprotective transcription factor Nrf2 counteracts these detrimental effects by serving as a negative regulator of NF-κB signaling and promoting the transcription of antioxidant genes. Among these, GPX4 inhibits lipid peroxidation and suppresses ferroptosis, while HO-1 reduces oxidative stress. Furthermore, multiple microRNAs (miRNAs) modulate these interconnected pathways: miR-26a targets Trpc3 and Trpc6, thereby regulating SOCE [125]; miR-34a-5p controls the Nrf2/HO-1 axis [16]; miR-216b directly downregulates MAP2K6, inhibiting MAPK signaling [17]; and miR-15b regulates apoptosis by targeting Bcl-2 [135]. Collectively, these findings highlight ferroptosis and its regulatory networks as potential therapeutic targets in pancreatitis.

Phytochemicals derived from medicinal plants are widely regarded as promising candidates for novel drug discovery and therapeutic development due to their minimal toxicity and generally negligible side effects [21,22]. Numerous preclinical studies have demonstrated that phytochemicals and herbal formulations exert beneficial effects in the treatment of pancreatitis by targeting diverse molecular pathways [26,27]. Among these, calcium signaling has emerged as a central mechanism in pancreatitis pathogenesis, and several phytochemicals have been shown to mitigate acute and severe acute pancreatitis (AP and SAP) by modulating calcium homeostasis through different strategies. For instance, caffeine suppresses intracellular calcium release by inhibiting IP_3_Rs [140], while resveratrol enhances calcium efflux by stimulating Ca^2+^–Mg^2+^–ATPase and Ca^2+^–ATPase activity [198]. More recently, urolithin A was reported to attenuate ER–ER–mitochondria Ca^2+^ transfer by downregulating IP_3_R and VDAC1 expression [59].

Both MAPK and NF-κB pathways are considered crucial contributors to the pancreatitis pathophysiology. NF-κB is a transcription factor that acts as a master regulator of the transcription of many proinflammatory cytokines [91,157]. Meanwhile, MAPK controls different downstream transduction signals, including inflammatory, apoptotic, oxidative, and fibrotic signals [109,111,115]. Notably, crosstalk between NF-κB and MAPK amplifies pancreatitis inflammation and accelerates the conversion of local inflammation into systemic inflammation during AP. Additionally, the persistent activation of these pathways promotes both inflammation and fibrosis in CP. Activated P38 MAPK phosphorylates a downstream kinase called MK2, stimulating the NF-κB pathway [110]. NF-κB induces transcription of proinflammatory cytokines such as TNF-α and IL-1, which in turn activate MAPK, forming a positive feedback loop that ultimately exacerbates the inflammatory response [109]. Several upstream kinases modulate the function of NF-κB, such as Akt [95], PAK1 [157], and GSK-3β [102]. Notably, previous studies suggest that MAPK signaling exerts a dual role in the progression of chronic pancreatitis (CP), where its inhibition may either confer protection or exacerbate disease severity [114,115].

Over the last two decades, the MAPK and NF-κB pathways have been extensively studied in numerous studies aimed at examining the anti-inflammatory effects of various phytochemicals. Nanocurcumin exhibited anti-inflammatory action by inhibiting the TLR4/NF-κB signaling pathway [9]. Meanwhile, a recent in vitro and in vivo AP study reported that myricetin modulates two pathways: enhancing the anti-inflammatory axis AMPK-SIRT1 and suppressing the proinflammatory axis CaMKIV-IκBα, ultimately inhibiting NF-κB [94]. Additionally, colchicine has been approved by the FDA for treating other diseases [227,228] and has been shown to possess anti-inflammatory, antioxidant, and anti-apoptotic activities in NaT-induced SAP-ALI via modulating the expression of NF-κB, Nrf2, STAT3, and AKT [201]. Calycosin indirectly inhibits NF-κB by suppressing p38 MAPK phosphorylation, which acts as a positive upstream regulator of NF-κB [187]. Stigmasterol and ligustrazine mitigate the Erk pathway, resulting in shifting acinar cell death from necrosis to controlled apoptosis, inhibiting systemic inflammation induced by acinar cell necrosis, and alleviating AP at the early stage [191,192].

Oxidative stress plays a central role in driving injury in both the pancreas and extra-pancreatic organs [229,230]. Cytoplasmic Ca^2+^ overload leads to mitochondrial Ca^2+^ accumulation and excessive reactive oxygen species (ROS) production, which in turn triggers lipid peroxidation, increases cell membrane permeability, and further exacerbates Ca^2+^ dysregulation [231,232]. The Nrf2 signaling pathway functions as a key protective mechanism against oxidative stress and inflammation. It mitigates both [79]. Importantly, oxidative stress and inflammation are tightly interconnected: ROS activates NF-κB signaling to promote inflammation, whereas Nrf2 suppresses this response by enhancing the degradation of IκB proteins [79,80]. Given this crosstalk, Nrf2 is considered one of the major pathways of interest in studies evaluating the protective effects of phytochemicals in pancreatitis. Several phytochemicals—including visnagin, micheliolide, and galangin—have been shown to alleviate pancreatitis by activating Nrf2 while suppressing NF-κB [27,89,158]. More recently, kaempferol was incorporated into a mitochondria-targeted nanosystem designed to improve solubility, bioavailability, and safety. This formulation effectively alleviated severe acute pancreatitis (SAP) by activating the Nrf2/HO-1 pathway and elevating intracellular glutathione (GSH) levels [205].

Overproduction of ROS, or impairment of the antioxidant systems, alongside iron overload, results in ferroptosis, which is an iron-dependent lipid peroxidation of membrane phospholipids [62]. Recent studies have highlighted the suppression of ferroptosis as a potential therapeutic strategy for pancreatitis, possibly through enhancing antioxidant enzymes such as GPX4, promoting iron efflux or sequestration, and reducing pro-ferroptotic proteins like ACSL4. For instance, wedelolactone has been shown to activate the anti-ferroptotic enzyme GPX4, thereby protecting against acute pancreatitis [151]. Similarly, silibinin upregulates FTH1, which sequesters Fe^2+^, while simultaneously downregulating ACSL4 expression, collectively attenuating ferroptosis [70]. Recently, Neferine has been shown to bind to the Cys-288 residue of Keap1, thereby activating the Nrf2 signaling pathway and inducing transcription of the iron exporter FPN, which enhances cellular iron efflux [152].

MicroRNAs (miRNAs) are small, non-coding RNA molecules that regulate gene expression at the post-transcriptional level [13,14]. By modulating key pathways, including inflammatory, oxidative, and cytoprotective responses, miRNAs represent potential therapeutic targets in pancreatitis. Several phytochemicals have been reported to regulate miRNA expression in models of acute pancreatitis (AP) and severe acute pancreatitis (SAP), thereby exerting anti-inflammatory effects, for example, quercetin [17], salidroside [206], and emodin [132], as well as antioxidant effects, such as those induced by baicalin [197] and pinocembrin [16].

Chronic pancreatitis (CP) is a progressive fibroinflammatory disorder characterized by irreversible pancreatic damage. Progressive fibrosis represents the final pathological outcome of CP, with pancreatic stellate cell (PSC) activation recognized as an early cellular event driving fibrogenesis. Puerarin has been shown to upregulate GFAP, a marker of quiescent PSCs, while suppressing activated PSC markers such as Col1α1, α-SMA, and fibronectin [115]. More recently, curcumin was reported to inhibit PSC activation and collagen deposition by activating the Nrf2/HO-1 pathway, thereby attenuating pancreatic fibrosis in a CP mouse model [26]. In addition, nanocurcumin has demonstrated protective effects in preclinical models of acute pancreatitis [98] and was subsequently evaluated in a double-blind, parallel-arm randomized controlled trial, which concluded that nanocurcumin can be safely administered as an adjuvant therapy in patients with acute pancreatitis [210].

Optimizing the therapeutic potential of phytochemicals and guiding future drug development strategies requires a detailed understanding of their pharmacokinetic properties. Unlike conventional small-molecule drugs, many phytochemicals face limitations related to solubility, stability, metabolism, and transport, which may restrict their clinical applicability despite promising biological effects.

A rigorous framework for evaluating the drug-likeness and oral bioavailability of phytochemicals integrates multiple parameters, including Lipinski’s Rule of Five (Ro5), predicted intestinal absorption, Caco-2 cell permeability assays, and interactions with efflux transporters such as P-glycoprotein (Pgp). Compounds that meet these criteria are more likely to exhibit favorable absorption and systemic exposure following oral administration. For instance, small, lipophilic molecules with high membrane permeability, such as caffeine and resveratrol, demonstrate efficient intestinal uptake and hence show promise as orally active interventions in pancreatitis models.

In contrast, larger or highly polar phytochemicals, while often biologically potent, tend to suffer from poor absorption, extensive first-pass metabolism, or active efflux, thereby limiting their systemic bioavailability. For such compounds, advanced formulation strategies may be necessary to enhance their clinical utility. Approaches include prodrug design, which improves lipophilicity or stability before metabolic conversion into the active form, as well as nanotechnology-based systems such as nanoencapsulation, liposomes, micelles, or solid lipid nanoparticles, which can enhance solubility, protect from premature degradation, and facilitate targeted delivery.

**Figure 5:**
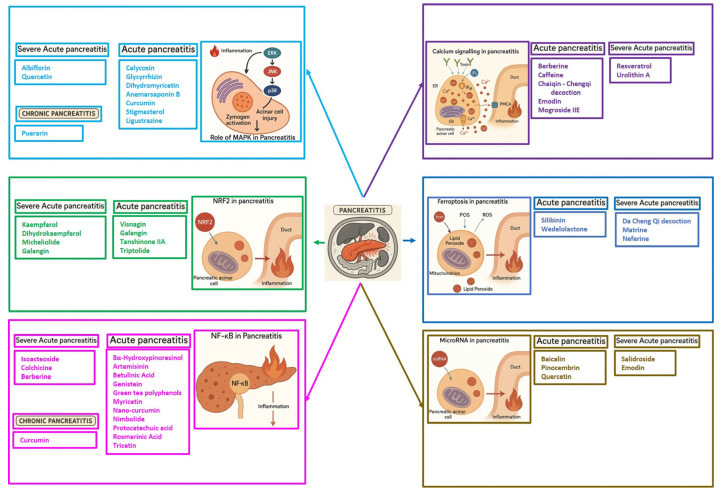
Overview of molecular targets modulated by phytochemicals. Schematic overview linking major pathogenic axes in pancreatitis to plant-derived modulators, grouped by disease severity. The central panel depicts the injured pancreas; colored spokes lead to six mechanisms with representative agents that have shown protective/therapeutic effects in experimental models. AP = acute pancreatitis; SAP = severe acute pancreatitis; CP = chronic pancreatitis.

Taken together, these considerations emphasize the importance of pharmacokinetic profiling in the translation of phytochemicals from preclinical promise to clinically viable therapies for pancreatitis. A systematic evaluation of their absorption, distribution, metabolism, and excretion (ADME) properties, coupled with innovative formulation technologies, may not only optimize their therapeutic potential but also accelerate the rational design of next-generation phytochemical-derived drugs. Frameworks, including Lipinski’s Rule of Five, P-glycoprotein interaction profiling, and bioavailability studies employing appropriate models, need to be established (Figure 5).

## 10. Conclusions

Pancreatitis remains a challenging disease with limited therapeutic options, where current management is largely supportive. Emerging evidence highlights the critical role of dysregulated calcium signaling, oxidative stress-induced ferroptosis, and microRNA networks in driving acinar cell injury, inflammation, and fibrogenesis. Phytochemicals—spanning flavonoids, alkaloids, terpenoids, and polyphenols—exert protective effects through multi-targeted actions, including modulation of NF-κB, Nrf2/HO-1, and ferroptosis-related pathways, as well as regulation of microRNAs. These natural compounds not only reduce oxidative stress and inflammatory cascades but also help restore cellular homeostasis, thereby offering promise as adjunctive or novel therapeutic strategies. Nevertheless, the translation of these preclinical findings into clinical practice is hindered by challenges in bioavailability, stability, and pharmacokinetics. Advanced drug delivery systems and rigorous pharmacokinetic profiling—including Lipinski’s Rule of Five, intestinal absorption models, and efflux transporter interactions—are crucial to optimize their therapeutic potential. Future research should focus on well-designed clinical trials, nanotechnology-driven formulations, and integrative pharmacological approaches to bridge the gap between laboratory efficacy and clinical applicability. In conclusion, phytochemicals represent a valuable reservoir of therapeutic candidates for acute, severe acute, and chronic pancreatitis, with the potential to shift management from symptomatic care toward disease-modifying interventions.

## Figures and Tables

**Table 7:** Phytochemical–physicochemical properties and pharmacokinetic properties of natural ligands for pancreatitis.

Phytochemical Name	Structure	Molecular Weight g/mol	H-Bond Acceptors	H-Bond Donors	LogP	Caco2 Permeability	Intestinal Absorption (Human) %	Skin Permeability (Log Kp)	P-Glyco Protein Substrate	P-Glyco Protein Inhibitor I/II
Acute pancreatitis										
8α-Hydroxypinoresinol	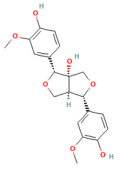	374.389	7	3	2.3051	0.342	80.934	−2.759	Yes	No/No
Albiflorin	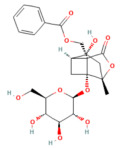	480.466	11	5	−1.5149	0.573	50.877	−2.737	Yes	No/No
Anemarsaponin B	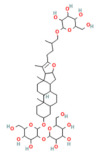	903.069	18	11	−0.8299	−0.975	0	−2.735	Yes	Yes/No
Artemisinin	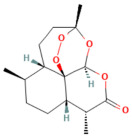	282.336	5	0	2.3949	1.295	97.543	−3.158	No	No/No
Baicalin	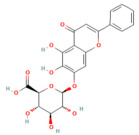	446.364	10	6	0.142	−0.67	26.224	−2.745	Yes	No/No
Berberine	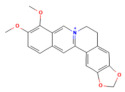	336.367	4	0	3.096	1.734	97.147	−2.576	Yes	No/Yes
Betulinic acid	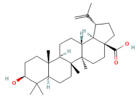	456.711	2	2	7.0895	1.175	99.763	−2.735	No	No/No
Caffeine	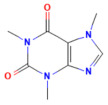	194.194	6	0	−1.0293	1.115	99.272	−3.376	No	No/No
Calycosin	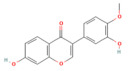	284.267	5	2	2.8798	0.96	95.098	−2.747	Yes	No/No
Chlorogenic acid	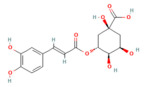	354.311	8	6	−0.645	−0.84	36.377	−2.735	Yes	No/No
Curcumin	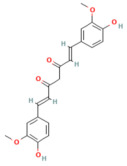	368.385	6	2	3.369	−0.093	82.19	−2.764	Yes	Yes/Yes
Dihydrodiosgenin	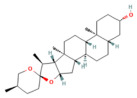	416.646	3	1	5.793	1.301	95.856	−2.973	No	Yes/Yes
Dihydromyricetin	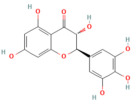	320.253	8	6	0.891	0.111	58.92	−2.735	Yes	No/No
Emodin	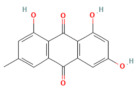	270.24	5	3	1.8872	0.055	74.485	−2.737	Yes	No/No
Galanin Like peptide	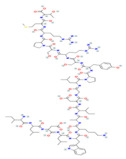	2332.76	31	33	8.184	−2.401	0	−2.735	Yes	No/No
Genistein	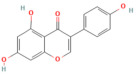	302.238	7	5	1.988	−0.229	77.207	−2.735	Yes	No/No
Glycyrrhizin	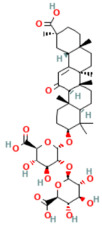	822.942	13	8	2.2456	−0.769	0	−2.735	Yes	No/No
Ligustrazine	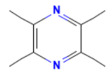	136.198	2	0	1.7102	1.805	98.076	−2.639	No	No/No
Mogroside IIE	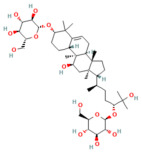	801.024	14	10	1.119	−0.76	11.46	−2.735	Yes	Yes/No
Myricetin	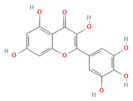	318.237	8	6	1.693	0.095	65.93	−2.735	Yes	No/No
Nimbolide	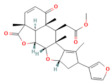	466.53	7	0	3.7431	0.92	100	−3.599	No	Yes/Yes
Pinocembrin	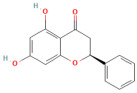	256.257	4	2	2.804	1.1152	92.417	−2.808	Yes	No/No
Protocatechuic acid	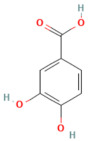	154.121	3	3	0.796	0.49	71.174	−2.727	No	No/No
Quercetin	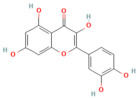	302.238	7	5	1.988	−0.229	77.20	−2.735	Yes	No/No
Resveratrol	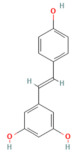	228.247	3	3	2.973	1.17	90.935	−2.737	Yes	No/No
Rosmarinic acid	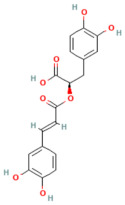	360.318	7	5	1.761	−0.937	32.516	−2.735	Yes	No/No
Salidroside	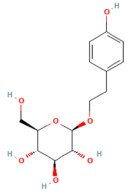	300.307	7	5	−1.248	0.46	45.49	−2.796	No	No/No
Silibinin	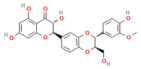	482.441	10	5	2.3627	0.435	61.861	−2.735	Yes	Yes/Yes
Stigmasterol	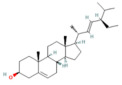	412.702	1	1	7.800	1.213	94.97	−2.783	No	Yes/Yes
Tanshinone IIA	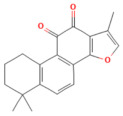	294.25	3	0	4.2479	1.419	96.253	−2.591	No	No/No
Tricetin	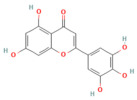	302.238	7	5	1.988	−0.272	78.366	−2.735	Yes	No/No
Triptolide	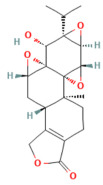	360.406	6	1	1.1031	0.401	83.195	−3.156	No	No/No
Visnagin	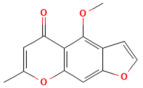	230.219	4	0	2.85	1.292	97.19	−2.264	No	No/No
Wedelolactone	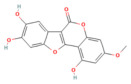	314.249	7	3	2.	−0.23	93.753	−2.735	Yes	No/No
Severe acute pancreatitis										
Berberine	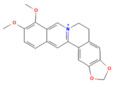	336.367	4	0	3.0963	1.734	97.147	−2.576	Yes	No/Yes
Colchicine	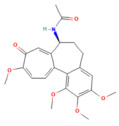	399.443	6	1	2.8716	1.139	97.245	−2.927	Yes	Yes/Yes
Dihydrokaempferol	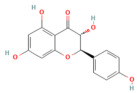	288.255	6	4	1.4807	0.996	59.072	−2.735	Yes	No/No
Emodin	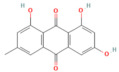	270.24	5	3	1.8872	0.055	74.485	−2.737	Yes	No/No
Galangin	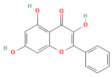	270.24	5	3	2.5868	0.999	93.985	−2.735	Yes	No/No
Isoacteoside	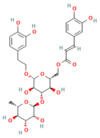	624.592	15	9	−1.0159	−1.391	21.587	−2.735	Yes	No/No
Isorhamnetin	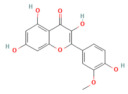	316.265	7	4	2.291	0.003	76.014	−2.735	Yes	No/No
Kaempferol	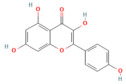	286.239	6	4	2.2824	0.032	74.29	−2.735	Yes	No/No
Matrine	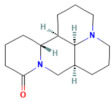	248.37	2	0	1.8717	1.436	94.897	−2.882	Yes	No/No
Micheliolide	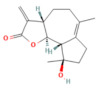	248.322	3	1	2.3555	1.291	96.495	−3.446	No	No/No
Neferine	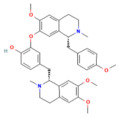	624.778	8	1	6.7623	0.368	88.197	−2.735	Yes	Yes/Yes
Quercetin	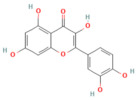	302,238	7	5	1.988	−0.229	77.207	−2.735	Yes	No/No
Resveratrol	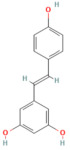	228.247	3	3	98.911	1.17	90.935	−2.737	Yes	No/No
Salidroside	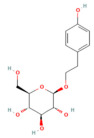	300.307	7	5	−1.2488	0.46	45.49	−2.796	No	No/No
Urolithin A	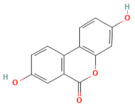	228.203	4	2	2.3574	1.061	93.918	−2.777	Yes	No/No
Chronic pancreatitis										
Catechin hydrate	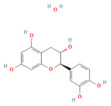	308.286	6	5	0.7214	0.159	63.919	−2.735	Yes	No/No
Curcumin	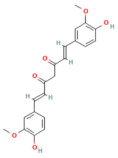	368.385	6	2	3.3699	−0.093	82.19	−2.764	Yes	Yes/Yes
Puerarin	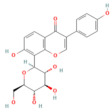	416.382	9	6	0.3861	0.223	67.446	−2.735	Yes	No/No

## Data Availability

No new data were created or analyzed in this study. Data sharing is not applicable to this article.

## Abbreviations

The following abbreviations are used in this manuscript:

Ach	Acetylcholine
ACSL4	Acyl-CoA synthetase long-chain family member 4
ALOX	Arachidonate lipoxygenase
AMPK	AMP-activated protein kinase
ANAPC13	Anaphase-promoting complex subunit 13
ANP	Acute necrotizing pancreatitis
AP	Acute pancreatitis
AR42J	Rat pancreatic acinar-like cell line
BAPTA	1,2-Bis(o-aminophenoxy)ethane-N,N,N′,N′-tetraacetic acid (Ca^2+^ chelator)
BMSCs	Bone marrow mesenchymal stem cells
Caco-2	Human intestinal epithelial cell line used for permeability assays
CAE	Caerulein
cADPR	Cyclic ADP-ribose
CCK	Cholecystokinin
CCK	Cholecystokinin receptor type
CCN2	Cellular communication network factor 2
CD36	Cluster of differentiation 36
CDE	Choline-deficient, ethionine-supplemented (diet)
COX5A	Cytochrome c oxidase subunit 5A
CP	Chronic pancreatitis
CRAC	Ca^2+^ release-activated Ca^2+^ channel
Cyt c	Cytochrome c
DCQD	Da Cheng Qi decoction
DHODH	Dihydroorotate dehydrogenase
ECM	Extracellular matrix
ERK	Extracellular signal-regulated kinase
ERBB3	Receptor tyrosine kinase ErbB3/HER3
EVs	Extracellular vesicles
FAEE	Fatty acid ethyl ester
FAT10	HLA-F adjacent transcript 10
FoxO1	Forkhead box O1
FPN (SLC40A1)	Ferroportin (iron exporter)
FSP1 (AIFM2)	Ferroptosis suppressor protein 1
FTH1	Ferritin heavy chain 1
FTL	Ferritin light chain
GCLC/GCLM	Glutamate–cysteine ligase catalytic/modifier subunit
GCH1	GTP cyclohydrolase 1
GPX4	Glutathione peroxidase 4
GR	Glutathione reductase
Grp75	Mitochondrial chaperone mortalin/Grp75
GSK-3β	Glycogen synthase kinase-3 beta
GST	Glutathione S-transferase
GSH	Reduced glutathione
HIF-1α	Hypoxia-inducible factor-1 alpha
HMGB1	High-mobility group box-1
HO-1	Heme oxygenase-1
HSP	Heat-shock protein
HTGP	Hypertriglyceridemia-induced pancreatitis
IDH2	Isocitrate dehydrogenase 2 (mitochondrial)
IEP	Interstitial edematous pancreatitis
IKKβ	IκB kinase beta
IL-1β	Interleukin-1 beta
IL-6	Interleukin-6
iNOS	Inducible nitric oxide synthase
IP3	Inositol 1,4,5-trisphosphate
IP3R	Inositol 1,4,5-trisphosphate receptor
JNK	c-Jun N-terminal kinase
Keap1	Kelch-like ECH-associated protein 1
KO	Knockout
LPCAT3	Lysophosphatidylcholine acyltransferase 3
LPS	Lipopolysaccharide
MAP2K4/MAP2K6	MAPK kinase 4/6
MAPK	Mitogen-activated protein kinase
MCU	Mitochondrial Ca^2+^ uniporter
MEK	MAPK/ERK (MAP2K1/2)
miRNA	MicroRNA
MK2	MAPK-activated protein kinase 2
MOD	Multiple organ dysfunction
MPTP	Mitochondrial permeability transition pore
mTORC2	Mechanistic target of rapamycin complex 2
MNCX/NCLX	Mitochondrial Na^+^/Ca^2+^ exchanger
MyD88	Myeloid differentiation primary response protein 88
NAADP	Nicotinic acid adenine dinucleotide phosphate
NaT	Sodium taurocholate
NFAT	Nuclear factor of activated T cells
NF-κB	Nuclear factor kappa-light-chain-enhancer of activated B cells
NLRP3	NOD-like receptor family pyrin domain-containing 3
NQO1	NAD(P)H quinone dehydrogenase 1
Nrf2	Nuclear factor erythroid 2-related factor 2
NP	Necrotizing pancreatitis
OA	Oleic acid
Orai1	Calcium release-activated calcium modulator 1
PAK1	p21-activated kinase 1
PAC	Pancreatic acinar cell
PDL	Pancreatic duct ligation
PE	Phosphatidylethanolamine
PGC-1α	Peroxisome proliferator-activated receptor-γ coactivator-1 alpha
PI3K	Phosphoinositide 3-kinase
PKC	Protein kinase C
PLA2	Phospholipase A2
PMCA	Plasma membrane Ca^2+^-ATPase
PPARγ	Peroxisome proliferator-activated receptor gamma
PTEN	Phosphatase and tensin homolog
PTGS2	Prostaglandin-endoperoxide synthase 2
PTK2	Protein tyrosine kinase 2
PUFA	Polyunsaturated fatty acid
PUFA-PL	PUFA-containing phospholipid
RELA	v-rel reticuloendotheliosis viral oncogene homolog A
Rictor	Rapamycin-insensitive companion of mTOR
RIP1/3	Receptor-interacting serine/threonine protein kinase 1/3
Ro5	Lipinski’s Rule of Five
ROS	Reactive oxygen species
RyR	Ryanodine receptor
SAP	Severe acute pancreatitis
SAP-ALI	Severe acute pancreatitis-associated acute lung injury
SARAF	SOCE-associated regulatory factor
SERCA	Sarco/endoplasmic reticulum Ca^2+^-ATPase
SIRT3/SIRT4	Sirtuin-3/Sirtuin-4
SLC3A2	Solute carrier family 3 member 2
SLC7A11	Cystine/glutamate antiporter light chain
SOCE	Store-operated Ca^2+^ entry
SOD1	Superoxide dismutase 1
STIM1	Stromal interaction molecule 1
TAK1	TGF-β-activated kinase 1
TBK1	TANK-binding kinase 1
TCA	Taurocholic acid
TGF-β	Transforming growth factor beta
TNF-α	Tumor necrosis factor alpha
TNFRSF1A	Tumor necrosis factor receptor superfamily member 1A
TLR4	Toll-like receptor 4
TRAF3/6	TNF receptor-associated factor 3/6
TRP	Transient receptor potential
TRPC3/TRPC6	Transient receptor potential canonical channels 3/6
TRPV	Transient receptor potential vanilloid
UCP2	Uncoupling protein-2
USP25	Ubiquitin-specific peptidase 25
VDAC1	Voltage-dependent anion channel 1
ZO-1	Zonula occludens-1
